# Outcome prediction by the 2022 European LeukemiaNet genetic-risk classification for adults with acute myeloid leukemia: an Alliance study

**DOI:** 10.1038/s41375-023-01846-8

**Published:** 2023-02-23

**Authors:** Krzysztof Mrózek, Jessica Kohlschmidt, James S. Blachly, Deedra Nicolet, Andrew J. Carroll, Kellie J. Archer, Alice S. Mims, Karilyn T. Larkin, Shelley Orwick, Christopher C. Oakes, Jonathan E. Kolitz, Bayard L. Powell, William G. Blum, Guido Marcucci, Maria R. Baer, Geoffrey L. Uy, Wendy Stock, John C. Byrd, Ann-Kathrin Eisfeld

**Affiliations:** 1 Clara D. Bloomfield Center for Leukemia Outcomes Research, The Ohio State University Comprehensive Cancer Center, Columbus, OH USA; 2 Alliance Statistics and Data Management Center, The Ohio State University Comprehensive Cancer Center, Columbus, OH USA; 3 The Ohio State University, Department of Internal Medicine, Columbus, OH USA; 4 Division of Hematology, Department of Internal Medicine, The Ohio State University Comprehensive Cancer Center, Columbus, OH USA; 5 Department of Genetics, University of Alabama at Birmingham, Birmingham, AL USA; 6 Division of Biostatistics, College of Public Health, The Ohio State University, Columbus, OH USA; 7 Monter Cancer Center, Hofstra Northwell School of Medicine, Lake Success, NY USA; 8 Wake Forest Baptist Comprehensive Cancer Center, Winston-Salem, NC USA; 9 Emory University School of Medicine, Atlanta, GA USA; 10 Department of Hematological Malignancies Translational Science, Gehr Family Center for Leukemia Research, City of Hope Medical Center and Beckman Research Institute, Duarte, CA USA; 11 University of Maryland Greenebaum Comprehensive Cancer Center, Baltimore, MD USA; 12 Washington University School of Medicine, St. Louis, MO USA; 13 Department of Medicine, University of Chicago, Chicago, IL USA; 14 Department of Internal Medicine, University of Cincinnati, Cincinnati, OH USA

**Keywords:** Cancer genetics, Risk factors, Cancer genetics, Cancer genetics

## Abstract

Recently, the European LeukemiaNet (ELN) revised its genetic-risk classification of acute myeloid leukemia (AML). We categorized 1637 adults with AML treated with cytarabine/anthracycline regimens according to the 2022 and 2017 ELN classifications. Compared with the 2017 ELN classification, 2022 favorable group decreased from 40% to 35% and adverse group increased from 37% to 41% of patients. The 2022 genetic-risk groups seemed to accurately reflect treatment outcomes in all patients and patients aged <60 years, but in patients aged ≥60 years, relapse rates, disease-free (DFS) and overall (OS) survival were not significantly different between intermediate and adverse groups. In younger African-American patients, DFS and OS did not differ between intermediate-risk and adverse-risk patients nor did DFS between favorable and intermediate groups. In Hispanic patients, DFS and OS did not differ between favorable and intermediate groups. Outcome prediction abilities of 2022 and 2017 ELN classifications were similar. Among favorable-risk patients, myelodysplasia-related mutations did not affect patients with *CEBPA*^bZIP^ mutations or core-binding factor AML, but changed risk assignment of *NPM1*-mutated/*FLT3*-ITD-negative patients to intermediate. *NPM1*-mutated patients with adverse-risk cytogenetic abnormalities were closer prognostically to the intermediate than adverse group. Our analyses both confirm and challenge prognostic significance of some of the newly added markers.

## Introduction

Pretreatment cytogenetic findings were first to be used to prognostically stratify patients with acute myeloid leukemia (AML) [1–9]. Subsequently, several gene mutations were demonstrated to provide additional prognostic information [10–19]. Therefore, in 2010, the first edition of the European LeukemiaNet (ELN) recommendations for diagnosis and management of AML included a standardized system for reporting cytogenetic findings and select gene mutations to enable meaningful comparisons among studies correlating genetic findings with clinical outcome [20]. Soon thereafter, several large studies demonstrated the ability of the 2010 ELN classification to prognostically separate the favorable and adverse groups from each other and from the two intermediate groups with regard to probability of complete remission (CR) attainment, disease-free (DFS) and overall survival (OS) [21–24]. This was shown to be independent from other prognostic factors by multivariable analyses [22]. The ELN classification was then modified in 2017 by combining two intermediate groups into one, recommending mutation analysis to be performed in all patients, not only those with cytogenetically normal AML (CN-AML), considering only biallelic *CEBPA* mutations as prognostically favorable, requiring determination of high and low allelic ratios for internal tandem duplications of *FLT3* (*FLT3*-ITD), and adding *ASXL1*, *RUNX1* and *TP53* mutations as adverse-risk markers [25]. The ELN genetic-risk classifications have been used in daily clinical practice to predict response to conventional chemotherapy and help guide treatment decisions, including the need for more intensive consolidation or alternative regimens.

The recently updated 2022 ELN recommendations include a revised genetic-risk classification that incorporates recent advances in our understanding of prognostic significance of genetic alterations in AML [26]. Major changes from 2017 ELN include the addition of seven myelodysplasia-related mutations to the adverse group (in the absence of favorable-risk markers); placing *NPM1*-mutated patients with adverse-risk cytogenetic abnormalities in the adverse group; consideration of only the presence, not allelic ratio, of *FLT3*-ITD; and the substitution of biallelic *CEBPA* mutations with in-frame mutations affecting the basic leucine zipper (bZIP) region of the *CEBPA* gene (*CEBPA*^bZIP^) as favorable-risk markers. Additionally, t(8;16)(p11.2;p13.3)/*KAT6A*::*CREBBP* and t(3;v)(q26.2;v)/*MECOM*(*EVI1*)-rearranged have been added to the adverse group, and hyperdiploid karyotypes with ≥3 trisomies without structural abnormality are no longer considered as complex [26].

The goals of our study were to assess how well the 2022 ELN genetic-risk groups associate with treatment response, DFS and OS, to compare the performance of the 2022 and 2017 ELN classifications both in all patients and in age cohorts, and to assess the effectiveness of newly introduced features in outcome prediction.

## Methods

### Patients and treatment

We analyzed 1637 adults diagnosed with de novo AML (other than acute promyelocytic leukemia, which is not included in the 2022 ELN guidelines), including 1040 patients aged <60 years (hereafter referred to as younger) and 597 patients aged ≥60 years (older), who were treated on Cancer and Leukemia Group B (CALGB) frontline treatment protocols between 1986 and 2013. CALGB is now part of the Alliance for Clinical Trials in Oncology (Alliance). For definition of race and ethnicity please see Supplementary Information. Younger patients received intensive cytarabine/daunorubicin-based induction chemotherapy and consolidation with high-dose chemotherapy or autologous hematopoietic stem-cell transplantation (HSCT; details of treatment trials are provided in the Supplementary Information). All patients aged ≥60 years received cytarabine/daunorubicin-based chemotherapy.

Analyses of DFS and OS were conducted on patients who did not receive an allogeneic HSCT in first CR (94% of patients; 6% underwent HSCT). All patients provided written informed consent to participate in treatment studies and for the research use of their specimens before enrollment in agreement with the Declaration of Helsinki. Study protocols were approved by the Institutional Review Board at each center.

### Cytogenetic and molecular genetic analyses

Cytogenetic analyses of pretreatment bone marrow (BM) and/or blood samples were performed by CALGB/Alliance-approved institutional laboratories and the results confirmed by central karyotype review [27]. CN-AML was determined by analysis of ≥20 metaphase cells from BM subjected to short-term (24–48-h) unstimulated cultures [27].

The mutational status of the *ASXL1*, *BCOR*, *EZH2*, *NPM1*, *RUNX1*, *SF3B1*, *SRSF2*, *STAG2*, *TP53*, *U2AF1* and *ZRSR2* genes was determined centrally at The Ohio State University in patients’ DNA extracted from viably frozen cells collected via companion protocol CALGB 20202 by targeted amplicon sequencing using the MiSeq platform (Illumina, San Diego, CA) [28]. Testing for *FLT3*-ITD was done using the Sanger sequencing method [13]. Detection of *CEBPA*^bZIP^ mutations was performed according to the 2022 ELN guidelines [26] using mutational profiling via targeted amplicon sequencing and/or transcriptional profiling [29]. Further experimental details are provided in the Supplementary Information.

### Clinical endpoints and statistical analysis

Clinical endpoints were defined according to generally accepted criteria [20, 25, 26, 30] and treatment study protocols.

DFS was measured from the date of CR until the date of relapse or death from any cause, and relapse-free patients were censored at the last follow-up. OS was measured from the date on study until the date of death, and patients alive at last follow-up were censored. Data quality was ensured by review of data by the Alliance Statistics and Data Management Center and by the chairpersons of included studies following Alliance policies.

Pretreatment characteristics were compared using the Fisher’s exact and Wilcoxon rank-sum tests for categorical and continuous variables, respectively. For time-to-event analyses, we calculated survival estimates using the Kaplan-Meier method and compared groups using the log-rank test [31]. For comparisons of predictive values of the 2022 and 2017 ELN genetic-risk classifications, we used receiver operating characteristic (ROC) curves as graphical plots. The areas under the curve (AUC) are provided together with the 95% confidence intervals. An AUC = 0.50 denotes lack of prediction ability, equivalent to that of random chance or a coin flip, whereas an AUC = 1.00 (highest possible value) indicates perfect prediction ability. Commonly accepted criteria are that an AUC of 0.6–0.69, 0.7–0.79, 0.8–0.89, and ≥0.9 indicate, respectively, poor, fair, good and very good prediction ability [32, 33]. In our models with a binary outcome, the AUC is equal to the more commonly known c-statistic [31–34]. The binary outcomes we considered were CR achievement (yes/no), relapse (yes/no), DFS (relapsed or dead versus relapse-free at three years) and OS (dead or alive at three years).

The dataset was locked on August 24, 2022. Data collection and statistical analyses were performed by the Alliance Statistics and Data Management Center using SAS 9.4 and TIBCO Spotfire S + 8.2. The median follow-up for patients still alive was 7.6 years.

## Results

### The distribution of risk-groups according to the 2022 and 2017 ELN classifications

**Fig. 1:**
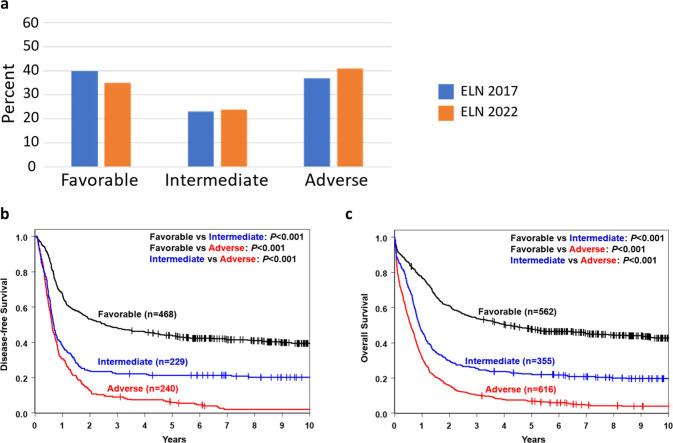
Distribution of the genetic-risk groups and outcome of all patients with de novo acute myeloid leukemia categorized into the three genetic-risk groups according to the 2022 European LeukemiaNet (ELN) recommendations. **a** Bar charts depicting the distribution of the genetic-risk groups in all patients categorized according to the 2022 ELN classification and of those grouped according to the 2017 ELN guidelines. **b** Disease-free survival and **c** overall survival of all patients classified according to the 2022 ELN guidelines.

Per the 2022 ELN classification, 35% of patients were assigned to the favorable group, 24% to intermediate and 41% to the adverse group. This represents a decrease in the proportion of patients in the favorable group and increase in the proportion of patients in the adverse group compared with the respective genetic-risk groups using 2017 ELN classification, namely, 40%, 23%, and 37% (Fig. 1a). More detailed reallocation of patients from 2017 ELN genetic-risk groups into the 2022 ELN ones is illustrated by Supplementary Fig. 1.

### Associations between 2022 ELN genetic-risk groups and clinical outcome

Eighty-four percent of favorable-risk patients achieved a CR, compared with 68% of intermediate-risk and 44% of adverse-risk patients (*P* < 0.001, Table 1). Relapse rates also differed among genetic-risk groups (52% vs 72% vs 87%, *P* < 0.001) as did DFS (5-year rates, 44% vs 21% vs 6%, *P* < 0.001; Fig. 1b) and OS (5-year rates, 48% vs 22% vs 7%, *P* < 0.001; Fig. 1c; Table 1). Multivariable analyses revealed that CR rates, DFS and OS remained better for patients in the favorable and intermediate groups compared with patients in the adverse group (*P* < 0.001 for all comparisons) after adjustment for such established prognostic factors in AML as age, WBC or platelets (Supplementary Table 1). Thus, the 2022 ELN genetic-risk groups seemed to accurately reflect patient outcomes with respect to achievement of CR, relapse rates, DFS and OS. However, when we compared the abilities of outcome prediction between the 2022 and 2017 ELN classifications, we found no significant differences with regard to CR achievement [area under the curve (AUC), 0.70 vs 0.71, *P* = 0.44], relapse rates (AUC, 0.67 vs 0.67, *P* = 0.74); DFS (AUC, 0.71 vs 0.70, *P* = 0.74) and OS (AUC, 0.74 vs 0.73, *P* = 0.15; Supplementary Fig. 2a–d), indicating that the 2022 ELN genetic-risk classification does not represent a clinically relevant increment of improvement over the 2017 ELN classification for outcome prediction in our adult patients with AML aged 17–89 years.

**Table 1:** Pretreatment characteristics and treatment outcome of patients with AML categorized according to the 2022 ELN genetic-risk classification.

Characteristic	Favorable *n* = 580 (I)	Intermediate *n* = 390 (II)	Adverse *n* = 667 (III)	*P*^a^ I vs II	*P*^a^ I vs III	*P*^a^ II vs III
Age, years				0.38	<0.001	<0.001
Median	50	50	61			
Range	17–84	17–85	17–89			
Age group, *n* (%)				0.14	<0.001	<0.001
Younger	439 (76)	278 (71)	323 (48)			
Older	141 (24)	112 (29)	344 (52)			
Sex, *n* (%)				0.02	0.001	<0.001
Male	310 (53)	179 (46)	417 (63)			
Female	270 (47)	211 (54)	250 (37)			
Race, *n* (%)				0.84	1.00	0.77
White	497 (88)	339 (88)	572 (87)			
Non-White	71 (13)	46 (12)	83 (13)			
Hemoglobin, g/dl				0.29	0.73	0.15
Median	9.2	9.3	9.2			
Range	2.3–25.1	2.9–15.0	3.0–15.8			
Platelet count, ×10^9^/l				0.006	0.09	0.27
Median	49	57	55			
Range	6–648	7–850	4–989			
WBC count, ×10^9^/l				0.004	<0.001	<0.001
Median	26.6	30.3	12.9			
Range	0.4–355.0	0.6–475.0	0.1–560.0			
Blood blasts, %				<0.001	<0.001	<0.001
Median	50	65	41			
Range	0–97	0–99	0–99			
Bone marrow blasts, %				<0.001	0.77	<0.001
Median	63	74	63			
Range	0–97	0–99	5–99			
Extramedullary involvement, *n* (%)	173 (31)	93 (25)	131 (21)	0.06	<0.001	0.10
2017 ELN, *n* (%)				<0.001	<0.001	<0.001
Favorable	551 (95)	70 (18)	40 (6)			
Intermediate	16 (3)	276 (71)	78 (12)			
Adverse	13 (2)	44 (11)	549 (82)			
Complete remission rate, *n* (%)^b^	485 (84)	264 (68)	291 (44)	<0.001	<0.001	<0.001
Relapse rate, *n* (%)	245 (52)	166 (72)	209 (87)	<0.001	<0.001	<0.001
Disease-free survival				<0.001	<0.001	<0.001
Median, years	2.6	0.7	0.6			
% Disease-free at 1 year (95% CI)	67 (62–71)	38 (32–44)	31 (25–37)			
% Disease-free at 3 years (95% CI)	48 (43–53)	22 (17–28)	9 (6–13)			
% Disease-free at 5 years (95% CI)	44 (39–48)	21 (16–27)	6 (4–10)			
Overall survival				<0.001	<0.001	<0.001
Median, years	4.1	0.9	0.6			
% Alive at 1 year (95% CI)	77 (73–80)	47 (41–52)	32 (28–35)			
% Alive at 3 years (95% CI)	54 (50–58)	26 (21–30)	10 (8–13)			
% Alive at 5 years (95% CI)	48 (44–52)	22 (18–27)	7 (5–9)			

^a^*P*-values are from the Fisher’s exact test for categorical variables, Wilcoxon rank-sum test for continuous variables and the log rank test for disease-free and overall survival and are for the specified two-way comparisons.

^b^For complete remission (CR) analyses the denominator included patients who received an allogeneic hematopoietic stem-cell transplantation in first CR. Relapse rate, disease-free and overall survival analyses exclude patients who received an allogeneic hematopoietic stem-cell transplantation in first CR (favorable: *n* = 562, those who achieved a CR *n* = 468; intermediate: *n* = 355, those who achieved a CR *n* = 229; adverse: *n* = 616. those who achieved a CR *n* = 240).

### Breakdown of genetic-risk groups in younger and older patients for 2022 and 2017 ELN classifications and associations with outcome

**Fig. 2:**
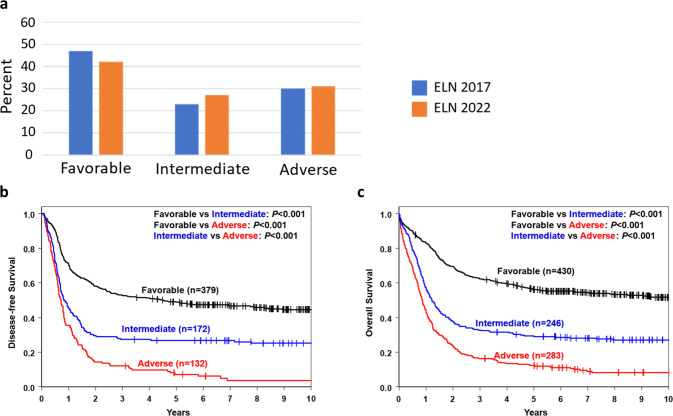
Distribution of the genetic-risk groups and outcome of younger adults under the age of 60 years with de novo acute myeloid leukemia categorized into the three genetic-risk groups according to the 2022 European LeukemiaNet (ELN) recommendations. **a** Bar charts depicting the distribution of the genetic-risk groups in younger patients categorized according to the 2022 ELN classification and of those grouped according to the 2017 ELN guidelines. **b** Disease-free survival and **c** overall survival of younger patients classified according to the 2022 ELN guidelines.

**Fig. 3:**
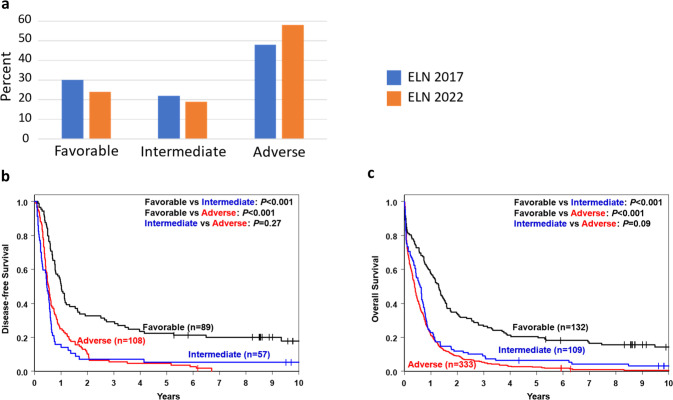
Distribution of the genetic-risk groups and outcome of older patients aged 60 years or older with de novo acute myeloid leukemia categorized into the three genetic-risk groups according to the 2022 European LeukemiaNet (ELN) recommendations. **a** Bar charts depicting the distribution of the genetic-risk groups in older patients categorized according to the 2022 ELN classification and of those grouped according to the 2017 ELN guidelines. **b** Disease-free survival and **c** overall survival of older patients classified according to the 2022 ELN guidelines.

It is well established that the distributions of many cytogenetic abnormalities and gene mutations differ between younger and older patients [19, 22, 28, 35–40], as did the distributions of genetic-risk groups and their associations with outcome in 2010 ELN [21, 22] and 2017 ELN [41–43] classifications. Moreover, the availability of new regimens for older and/or unfit patients makes assessment of the likelihood to respond to therapy separately in older and younger patients of importance. Thus, we assessed the risk-group distributions and outcomes of patients aged <60 years and those aged ≥60 years. As Figs. 2a and 3a illustrate, younger patients were more often classified in the favorable (42% vs 24%) and intermediate (27% vs 19%) 2022 ELN risk-groups than older patients, whereas the latter were more frequent in the adverse (58% vs 31%) risk-group. Compared with the 2017 ELN classification, the proportions of younger patients in the 2022 ELN favorable group decreased (42% vs 47%) and in intermediate group increased (27% vs 23%). Among older patients, the 2022 ELN criteria resulted in smaller favorable (24% vs 30%) and intermediate (19% vs 22%) groups and an enlarged adverse group (58% vs 48%), compared with the 2017 ELN classification.

Concerning treatment outcomes of younger patients, the 2022 ELN genetic-risk groups essentially associated with the expected outcomes, with the attainment of CR, relapse rates, DFS and OS differing significantly among the favorable, intermediate and adverse groups (Fig. 2b, c; Supplementary Table 2). Among patients aged ≥60 years, those in the favorable group had better outcome than patients in both remaining groups. However, with the exception of CR rates, which were higher for intermediate- than adverse-risk patients (54% vs 35% *P* < 0.001), the outcome of patients classified in the intermediate group was very poor and did not differ significantly from outcome of the adverse group with regard to relapse rates (89% vs 88% *P* = 1.00), DFS (5-year rates, 5% vs 5%, *P* = 0.27) or OS (5-year rates, 6% vs 2%, *P* = 0.09; Fig. 3b, c, Supplementary Table 3).

We also performed exploratory analyses of the outcomes of younger patients of African-American ancestry and those self-identifying as Hispanics. We found no significant differences in DFS of African-American patients between the 2022 ELN favorable and intermediate (5-year rates, 32% vs 30%, *P* = 0.42) groups nor in DFS (5-year rates, 30% vs 0%, *P* = 0.30) and OS (5-year rates, 24% vs 3%, *P* = 0.46) between intermediate and adverse groups (Supplementary Fig. 3a, b, Supplementary Table 4). Moreover, among younger Hispanic patients, we observed no significant differences in DFS (5-year rates, 47% vs 67%, *P* = 0.42) or OS (5-year rates, 61% vs 71%, *P* = 0.67) between the 2022 ELN favorable and intermediate groups (Supplementary Fig. 3c, d, Supplementary Table 5). There were not enough patients aged ≥60 years in either racial-ethnic group for similar analyses.

Next, we compared the abilities of outcome prediction between the 2022 ELN and 2017 ELN classifications separately in younger and older patients. We found no significant advantage for the use of 2022 ELN classification over the 2017 ELN one in either the younger (Supplementary Fig. 4a–d) or older patients (Supplementary Fig. 5a–d).

### Assessment of the newly introduced prognostic markers

#### AML with myelodysplasia-related gene mutations

**Fig. 4:**
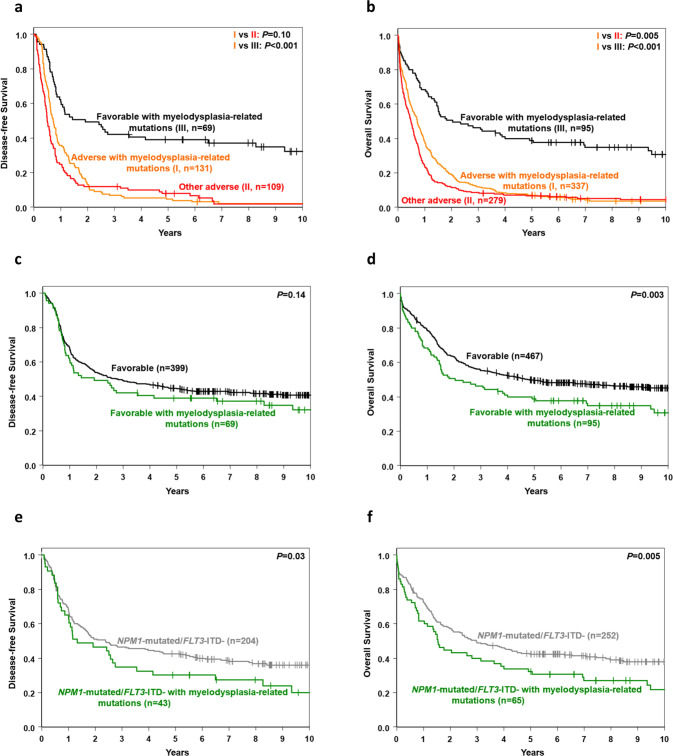
Outcomes of patients with de novo acute myeloid leukemia categorized according to the presence or absence of myelodysplasia-related mutations. **a** Disease-free survival and **b** overall survival of patients with myelodysplasia-related mutations with and those without favorable-risk AML subtypes, and of patients in the 2022 ELN adverse group who do not harbor myelodysplasia-related mutations. **c** Disease-free survival and **d** overall survival of patients in the 2022 ELN favorable group with myelodysplasia-related mutations and favorable-risk AML subtypes, and of patients in the 2022 ELN favorable group who do not have myelodysplasia-related mutations. **e** Disease-free survival and **f** overall survival of patients in the 2022 ELN favorable group with *NPM1* mutations, no *FLT3*-ITD and myelodysplasia-related mutations, and of patients in the 2022 ELN favorable group with *NPM1* mutations without *FLT3*-ITD or myelodysplasia-related mutations.

A major change in the 2022 ELN classification was the addition of seven myelodysplasia-related gene mutations (in the *BCOR*, *EZH2*, *SF3B1*, *SRSF2*, *STAG2*, *U2AF1* and *ZRSR2* genes) to the *ASXL1*, *RUNX1* and *TP53* mutations already included in the 2017 ELN classification as criteria for adverse group assignment, unless they co-exist with “favorable-risk AML subtypes” [26]. Indeed, in our cohort, patients with myelodysplasia-related mutations and no favorable genetic features had a low CR rate similar to the CR rate of patients with other adverse-risk markers (43% vs 44%, *P* = 0.75) and their DFS was not significantly different (5-year rates, 5% vs 8%, *P* = 0.10). However, patients with myelodysplasia-related mutations had longer OS (1-year rates, 38% vs 24%, 5-year rates, 7% vs 7%, *P* = 0.005). Conversely, the outcome of patients with myelodysplasia-related mutations without favorable-risk features was worse than the outcome of patients harboring myelodysplasia-related mutations together with favorable genetic-risk markers (CR rates, 43% vs 73%, *P* < 0.001; DFS, 5-year rates, 5% vs 39%, *P* < 0.001; OS, 5-year rates, 7% vs 39%, *P* < 0.001; Fig. 4a, b, Supplementary Table 6).

However, the outcome of patients with favorable-risk AML harboring myelodysplasia-related mutations was worse than the outcome of patients with favorable-risk AML without myelodysplasia-related mutations (CR rates, 73% vs 86%, *P* = 0.004; OS, 5-year rates, 39% vs 50%, *P* = 0.003), although the difference in DFS was not statistically significant (5-year rates, 39% vs 45%, *P* = 0.14; Fig. 4c, d, Supplementary Table 7).

Notably, the favorable genetic-risk group comprises three distinct subtypes, namely *NPM1*-mutated patients without *FLT3*-ITD, patients with core-binding factor AML (CBF-AML), and those with *CEBPA*^bZIP^ mutations. Thus, we next tested whether co-occurring myelodysplasia-related mutations impacted each of the aforementioned subsets alike. We found that co-occurring myelodysplasia-related mutations did not substantially affect the favorable impact of CBF-AML (CBF-AML with myelodysplasia-associated mutations vs CBF-AML without: CR rates, 93% vs 92%, *P* = 1.00; DFS, 5-year rates, 57% vs 51%, *P* = 0.65; OS, 5-year rates, 66% vs 63%, *P* = 0.65; Supplementary Table 8, Supplementary Fig. 6a, b), or of *CEBPA*^bZIP^ mutations (CR rates: 76% vs 85%, *P* = 0.33; DFS, 5-year rates, 38% vs 43%, *P* = 0.74; OS, 5-year rates: 38% vs 50%, *P* = 0.49; Supplementary Table 9, Supplementary Fig. 6c, d). However, the presence of myelodysplasia-related mutations resulted in worsening treatment outcome of *NPM1*-mutated/*FLT3*-ITD-negative patients, who had lower CR rates (67% vs 81%, *P* = 0.02), and shorter DFS (5-year rates, 30% vs 43%, *P* = 0.03) and OS (5-year rates, 32% vs 42%, *P* = 0.005) than *NPM1*-mutated/*FLT3*-ITD-negative patients without myelodysplasia-associated mutations (Fig. 4e, f, Supplementary Table 10). Consequently, we compared outcome of the *NPM1*-mutated/*FLT3*-ITD-negative patients who carried myelodysplasia-related mutations with outcome of patients included in the 2022 ELN intermediate group. Surprisingly, we found no significant differences between these subsets in CR rates (67% vs 68%, *P* = 1.00), relapse rates (70% vs 72%, *P* = 0.71), DFS (5-year rates, 30% vs 21%, *P* = 0.19) or OS (5-year rates, 32% vs 22%, *P* = 0.28; Supplementary Table 11). These results suggest that the prognostic significance of myelodysplasia-related mutations that “co-occur with favorable-risk AML subtypes” is not the same for all genetic subtypes comprising the 2022 ELN favorable group.

#### AML with NPM1 mutations co-occurring with adverse-risk cytogenetic features

**Fig. 5:**
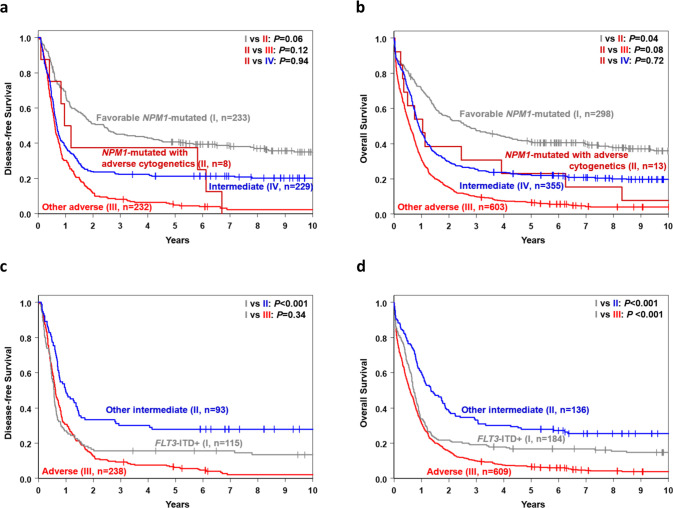
Outcomes of patients with de novo acute myeloid leukemia categorized according to the criteria newly introduced by the 2022 European LeukemiaNet recommendations. **a** Disease-free survival and **b** overall survival of patients with *NPM1* mutations and no *FLT3*-ITD categorized according to the presence or absence of adverse-risk cytogenetic features. Outcomes of patients classified in the 2022 ELN favorable and adverse (excluding *NPM1*-mutated patients with adverse-risk cytogenetic features) groups are shown for comparison. **c** Disease-free survival and **d** overall survival of intermediate-risk patients who harbor *FLT3*-ITD, compared with other patients included in the intermediate group and of patients in the adverse group.

Another refinement of the 2022 ELN genetic-risk classification was that the presence of adverse-risk cytogenetic abnormalities in *NPM1*-mutated AML now defines adverse risk [26]. Hence, we compared the outcome of *NPM1*-mutated patients classified in the favorable group (i.e., *NPM1*-mutated patients without *FLT3*-ITD) with outcome of *NPM1*-mutated/*FLT3*-ITD-negative patients with adverse-risk chromosome abnormalities. We found that although the CR rate of *NPM1*-mutated patients with adverse-risk abnormalities was lower than that of patients without adverse-risk abnormalities, the difference was not significant (64% vs 79%, *P* = 0.20), nor was the difference in relapse rates (88% vs 56%, *P* = 0.14). However, OS of *NPM1*-mutated patients with adverse-risk abnormalities was shorter (5-year rates, 23% vs 41%, *P* = 0.04) and DFS tended to be worse (5-year rates, 38% vs 41%, *P* = 0.06; Supplementary Table 12) than those of patients without adverse-risk cytogenetics. We then compared the former subset with other patients in the 2022 ELN adverse group. Although there were no significant differences for any of the endpoints analyzed, CR rates (*P* = 0.17), DFS (*P* = 0.12) and OS (*P* = 0.08), they all tended to be better for *NPM1*-mutated patients with adverse-risk abnormalities than for other adverse-risk patients (Supplementary Table 12). This prompted us to check whether the outcomes of *NPM1*-mutated patients with adverse-risk abnormalities were different from or similar to outcome of patients in the 2022 ELN intermediate group. As data in the Fig. 5a, b and Supplementary Table 13 show, *NPM1*-mutated patients with adverse-risk abnormalities were much closer prognostically to the 2022 ELN intermediate than adverse group.

Additionally, the 2022 ELN guidelines classify *NPM1*-mutated patients who harbor *FLT3*-ITD in the intermediate group, unless they also have adverse cytogenetics, which re-assigns such patients to the adverse group. Unfortunately, there were only four patients with *NPM1* mutations, *FLT3*-ITD and adverse cytogenetic abnormalities in our study, which precluded further analysis.

#### AML with FLT3-internal tandem duplication

AML with *FLT3*-ITD is now categorized in the intermediate group, irrespective of the allelic ratio or concurrent presence of *NPM1* mutations [26]. As this change was partially justified by the modifying impact of midostaurin-based therapy on *FLT3*-ITD without *NPM1* mutation, we separately analyzed the survival of patients treated with chemotherapy only and of those receiving midostaurin on the CALGB 10603 (RATIFY) protocol. Among patients receiving chemotherapy, those with *FLT3*-ITD had worse relapse rate (*P* = 0.01), DFS (*P* < 0.001) and OS (*P* < 0.001), and lower CR rates, but not significantly so (*P* = 0.08), than other patients included in the 2022 ELN intermediate group. Compared with the adverse group, although *FLT3*-ITD-positive patients had higher CR rates (*P* < 0.001) and longer OS (*P* < 0.001), their relapse rates (*P* = 0.21) and DFS (*P* = 0.34) were not significantly different (Fig. 5c, d, Supplementary Table 14). There were no significant differences in CR (*P* = 0.82) or relapse rates (*P* = 1.00), DFS (*P* = 0.14) or OS (*P* = 0.11) between a relatively small cohort of patients with *FLT3*-ITD treated with midostaurin and those receiving chemotherapy only (Supplementary Table 15, Supplementary Fig. 7a, b).

#### AML with CEBPA^bZIP^ mutations

One of the notable changes to the criteria used for classifying patients into the 2022 ELN favorable genetic-risk group was replacing biallelic *CEBPA* mutations by the in-frame *CEBPA*^bZIP^ mutations [26]. To assess this change, we first prepared Kaplan-Meier curves illustrating outcomes of all patients with biallelic *CEBPA* mutations (regardless whether they included *CEBPA*^bZIP^ mutations or not) and of all patients with *CEBPA*^bZIP^ mutations (irrespective whether these were monoallelic or biallelic mutations; Supplementary Fig. 8a, b). We could not formally compare these patient groups because some patients were included in both groups. Nevertheless, although OS of patients with *CEBPA*^bZIP^ mutations seemed slightly better, the improvement over biallelic *CEBPA* mutations did not appear compelling and the Kaplan-Meier curves representing DFS overlapped. We then compared outcomes of three subsets among *CEBPA*-mutated patients: those with monoallelic *CEBPA*^bZIP^ mutations, with biallelic *CEBPA*^bZIP^ mutations and with biallelic non-bZIP *CEBPA* mutations. As expected, patients in the last aforementioned group had the worst outcome. Somewhat surprisingly, patients with biallelic *CEBPA*^bZIP^ mutations had higher CR rates (95% vs 64%, *P* < 0.001), and longer OS (5-year rates, 53% vs 38%, *P* = 0.009), but not DFS (*P* = 0.27), than patients with monoallelic *CEBPA*^bZIP^ mutations (Supplementary Fig. 8c, d; Supplementary Table 16).

## Discussion

In this study of a large patient cohort with long follow-up, we applied the revised 2022 ELN criteria to stratify patients with de novo AML into genetic-risk groups and compared the performance of the modified classification with the previous one published in 2017. In the entire cohort, patients assigned to the 2022 ELN favorable group had better outcomes than those in the intermediate group, whose outcome was better than outcome of patients in the adverse group. Using ROC curves and the area under the curve we found that according to commonly accepted criteria [32, 33] the prediction ability of the 2022 ELN classification was fair for attainment of CR, DFS and OS, but poor for predicting relapse. Importantly, despite newly introduced modifications, the predictive ability of the 2022 ELN genetic-risk classification was essentially the same as predictive ability of the 2017 ELN classification with regard to all outcome endpoints tested.

Since the distribution of ELN genetic-groups differs between younger and older adults, with the former being more often classified in the favorable and the latter in the adverse groups, we analyzed patients aged <60 years and those aged ≥60 years separately. While in the younger patients the 2022 ELN classification separated genetic-risk groups quite well, among older adults only those in the favorable group had better outcomes, whereas there were no significant differences in relapse rates, DFS or OS between the intermediate and adverse groups. In a previous study, we observed a similar phenomenon in older patients classified according to the 2017 ELN criteria [42]. Therefore, our data support previous suggestions [22] that the ELN genetic-risk classification should be tailored to younger and older adults separately, which is also supported by the availability of treatment options targeting specific genetic alterations [44–48], whose incidence differs between younger and older patients [19, 22, 28, 35–43].

Moreover, our preliminary results indicate the need for large studies focused on racial/ethnic groups such as African-American and Hispanic patients. We found no significant differences in survival between 2022 ELN favorable and intermediate and between intermediate and adverse groups in patients of African-American ancestry, and no significant difference in DFS between favorable and intermediate groups in Hispanic patients. Although these results may be in part related to the relatively low number of patients we were able to analyze, previously identified racial/ethnic differences in the distribution of genetic alterations and outcomes [49–53] warrant application of 2022 ELN criteria to larger cohorts of African-American and Hispanic patients to confirm or refute our observations.

Among major aims of our study was evaluation of the new markers used for 2022 ELN group assignment. First, we found that patients harboring one or more myelodysplasia-related gene mutations without favorable-risk features had indeed very poor outcome placing them in the adverse group, and that the co-existence of favorable-risk features substantially improved patient outcomes. However, this improvement has not been sufficient to place the entire cohort of patients with favorable-risk AML and myelodysplasia-related mutations in the 2022 ELN favorable group, since their CR rates and OS were significantly worse. Importantly, the 2022 ELN favorable group is not homogeneous and consists of three major subsets. Our analyses revealed that myelodysplasia-related mutations did not negatively affect outcome of patients with CBF-AML and those harboring *CEBPA*^bZIP^ mutations, who would still be classified in the favorable group. However, the presence of myelodysplasia-related mutations in *NPM1*-mutated/*FLT3*-ITD-negative patients portended worse outcome, which placed these patients firmly in the 2022 ELN intermediate, not favorable, group. If these results are corroborated, future editions of the ELN classification should consider modification of risk-assignment for the aforementioned patient subsets.

Likewise, we have confirmed that patients with *NPM1* mutations co-occurring with adverse-risk cytogenetic features have worse prognosis than other *NPM1*-mutated patients included in the 2022 ELN favorable group. However, in contrast to the results of a recent study [54], the former subset seems to be closer prognostically to the 2022 ELN intermediate rather than adverse group. Unfortunately, the low number of patients with *NPM1* mutations and adverse-risk cytogenetics (*n* = 14) precludes us from making a definitive recommendation and shows the need for further study. Likewise, it is also necessary to confirm in larger patient cohorts that patients with *FLT3*-ITD belong to the intermediate group.

Based on recent reports [29, 55, 56], 2022 ELN recommendations replaced biallelic *CEBPA* mutations with the in-frame *CEBPA*^bZIP^ mutations as a criterion for classifying patients into the favorable genetic-risk group. We have generally confirmed that *CEBPA*^bZIP^ mutations confer better prognosis than biallelic, non-bZIP *CEBPA* mutations. Rather surprisingly, however, we found that patients with biallelic *CEBPA*^bZIP^ mutations had higher CR rates and longer OS than patients with monoallelic *CEBPA*^bZIP^ mutations, which differs from previously reported data [29, 55, 56]. The reasons for this discrepancy are unclear, but may be partially attributed to differences in treatment administered in different studies.

Among limitations of our study is its retrospective nature and the inability to assess the outcomes of patients with cytogenetic markers newly added to the 2022 ELN genetic-risk classification such as prognostically adverse t(8;16)(p11.2;p13.3)/*KAT6A*::*CREBBP* and t(3;v)(q26.2;v)/*MECOM*(*EVI1*)-rearranged, or hyperdiploid complex karyotypes with three or more trisomies without structural abnormalities that are no longer considered to be adverse markers. All these chromosome abnormalities occurred in too few patients for meaningful analyses in our study, thus warranting further collaborative efforts involving large AML study groups.

In summary, our large study assessing the newly revised 2022 ELN genetic-risk classification confirms its usefulness for prognostic stratification of patients with de novo AML. However, despite introduction of several new criteria, we found no advantage of this classification over the previous one published in 2017. Importantly, our data support earlier calls for separating younger from older adults because both the incidence of genetic alterations and outcomes differ between these age groups [19, 22, 28, 35–43]. It is hoped that future ELN recommendations will consider our and other [38, 57, 58] suggestions for refinement of the genetic-risk classification of AML.

## Supplementary information

Supplementary Information

## Data Availability

Patient data used in survival analyses were obtained from the Alliance Statistics and Data Management Center. Individual participant data will not be shared.
