# Supplementary material for: Outcome prediction by the 2022 European LeukemiaNet genetic-risk classification for adults with acute myeloid leukemia: an Alliance study
Source: Leukemia. 2023 Feb 23;37(4):788–98. doi: 10.1038/s41375-023-01846-8 (PMC10079544; doi:10.1038/s41375-023-01846-8)
Supplement: Supplementary file 1 — Supplementary Information [file 41375_2023_1846_MOESM1_ESM.docx]

**Supplementary Information**

**Outcome prediction by the 2022 European LeukemiaNet genetic-risk classification for adults with acute myeloid leukemia:**

**an Alliance study**

Short title: Outcome prediction according to 2022 ELN classification

Krzysztof Mrózek,^1,^* Jessica Kohlschmidt,^1,2,^* James S. Blachly,^3,4^ Deedra Nicolet,^1,2^

Andrew J. Carroll,^5^ Kellie J. Archer,^6^ Alice S. Mims,^3,4^ Karilyn T. Larkin,^1,3,4^

Shelley Orwick,^3,4^ Christopher C. Oakes,^3,4^ Jonathan E. Kolitz,^7^ Bayard L. Powell,^8^ William G. Blum,^9^ Guido Marcucci,^10^ Maria R. Baer,^11^ Geoffrey L. Uy,^12^ Wendy Stock,^13^ John C. Byrd^14,†^ and Ann-Kathrin Eisfeld^1,3,,4,†^

^1^ Clara D. Bloomfield Center for Leukemia Outcomes Research, The Ohio State University Comprehensive Cancer Center, Columbus, OH, USA.

^2^ Alliance Statistics and Data Management Center, The Ohio State University Comprehensive Cancer Center, Columbus, OH, USA.

^3^ The Ohio State University, Department of Internal Medicine, Columbus, OH, USA.

^4^ Division of Hematology, Department of Internal Medicine, The Ohio State University Comprehensive Cancer Center, Columbus, OH, USA.

^5^ Department of Genetics, University of Alabama at Birmingham, Birmingham, AL, USA.

^6^ Division of Biostatistics, College of Public Health, The Ohio State University, Columbus, OH,

USA.

^7^ Monter Cancer Center, Hofstra Northwell School of Medicine, Lake Success, NY, USA.

^8^ Wake Forest Baptist Comprehensive Cancer Center, Winston-Salem, NC, USA.

^9^ Emory University School of Medicine, Atlanta, GA, USA.

^10^ Department of Hematological Malignancies Translational Science, Gehr Family Center for

Leukemia Research, City of Hope Medical Center and Beckman Research Institute, Duarte,

CA, USA.

^11^ University of Maryland Greenebaum Comprehensive Cancer Center, Baltimore, MD, USA.

^12^ Washington University School of Medicine, St. Louis, MO, USA.

^13^ Department of Medicine, University of Chicago, Chicago, IL, USA.

^14^ Department of Internal Medicine, University of Cincinnati, Cincinnati, OH, USA.

* These authors contributed equally to this study.

^†^ These authors contributed equally to this study as senior authors.

**Participating institutions**

The following Cancer and Leukemia Group B (CALGB)/Alliance for Clinical Trials in Oncology (Alliance) institutions participated in this study and contributed five or more patients. For each institution, the current or last principal investigator and the cytogeneticists who analyzed the cases are listed as follows:

The Ohio State University Medical Center, Columbus, OH: Claire F. Verschraegen, Karl S. Theil, Diane Minka and Nyla A. Heerema; North Shore University Hospital, Manhasset, NY: Jonathan E. Kolitz, Prasad R. K. Koduru, Ayala Aviram-Goldring and Chandrika Sreekantaiah; Wake Forest University School of Medicine, Winston-Salem, NC: Heidi D. Klepin, P. Nagesh Rao, Wendy L. Flejter and Mark Pettenati; Roswell Park Cancer Institute, Buffalo, NY: Ellis G. Levine and AnneMarie W. Block; Dana Farber Cancer Institute, Boston, MA: Harold J. Burstein, Ramana V. Tantravahi, Cynthia C. Morton and Paola Dal Cin; Washington University School of Medicine, St. Louis, MO: Nancy L. Bartlett, Michael S. Watson, Eric C. Crawford, Jaime Garcia-Heras, Peining Li and Shashikant Kulkarni; University of Chicago Medical Center, Chicago, IL: Hedy L. Kindler, Diane Roulston, Katrin M. Carlson, Yanming Zhang and Michelle M. LeBeau; University of Iowa Hospitals, Iowa City, IA: Umar Farooq and Shivanand R. Patil; University of North Carolina, Chapel Hill, NC: Matthew I. Milowsky and Kathleen W. Rao; Duke University Medical Center, Durham, NC: Jeffrey Crawford, Sandra H. Bigner, Mazin B. Qumsiyeh, John Eyre and Barbara K. Goodman; Dartmouth Medical School, Lebanon, NH: Konstantin H. Dragnev, Doris H. Wurster-Hill and Thuluvancheri K. Mohandas; University of Maryland Greenebaum Cancer Center, Baltimore, MD: Heather D. Mannuel, Joseph R. Testa, Maimon M. Cohen, Judith Stamberg and Yi Ning; Ft. Wayne Medical Oncology/Hematology, Ft. Wayne, IN: Sreenivasa Nattam and Patricia I. Bader; Christiana Care Health Services, Inc., Newark, DE: Gregory A. Masters, Digamber S. Borgaonkar, Jeanne M. Meck, and Kathleen Richkind; Rhode Island Hospital, Providence, RI: Howard P. Safran, Teresita Padre-Mendoza, Hon Fong L. Mark, Shelly L. Kerman and Aurelia Meloni-Ehrig; Weill Medical College of Cornell University, New York, NY: Scott Tagawa, Ram S. Verma, Prasad R.K. Koduru, Andrew J. Carroll and Susan Mathew; University of Vermont Cancer Center, Burlington, VT: Peter A. Kaufman, Elizabeth F. Allen and Mary Tang;

Western Pennsylvania Hospital, Pittsburgh, PA: Gene G. Finley and Gerard R. Diggans; SUNY Upstate Medical University, Syracuse, NY: Stephen L. Graziano, Larry Gordon and Constance K. Stein; University of Massachusetts Medical Center, Worcester, MA: William V. Walsh, Philip L. Townes, Vikram Jaswaney, Kathleen Richkind, Patricia Miron and Michael J. Mitchell; Massachusetts General Hospital, Boston, MA: David Ryan, Leonard L. Atkins, Cynthia C. Morton and Paola Dal Cin; Eastern Maine Medical Center, Bangor, ME: Sarah J. Sinclair and Laurent J. Beauregard; Long Island Jewish Medical Center, Lake Success, NY: Jonathan E. Kolitz, Alan L. Shanske, Prasad R. K. Koduru, Ayala Aviram-Goldring and Chandrika Sreekantaiah; University of Alabama at Birmingham, Birmingham, AL: Robert Diasio and Andrew J. Carroll; Mount Sinai School of Medicine, New York, NY: Michael A. Schwartz and Vesna Najfeld; University of California San Diego Moores Cancer Center, San Diego, CA: Lyudmila A. Bazhenova, E. Robert Wassman, Jr., Renée Bernstein and Marie L. Dell'Aquila; University of Tennessee Cancer Center, Memphis, TN: Harvey B. Niell and Sugandhi A. Tharapel; University of Missouri/Ellis Fischel Cancer Center, Columbia, MO: Puja Nistala, Jeffrey R. Sawyer, Tim Hui-Ming Huang and Linda M. Pasztor; Walter Reed National Military Medical Center, Bethesda, MD: Karen G. Zeman, Rawatmal B. Surana, Digamber S. Borgaonkar, Karl S. Theil and Kathleen E. Richkind; University of Illinois, Chicago, IL: John G. Quigley, Maureen M. McCorquodale, Kathleen E. Richkind and Valerie Lindgren; Virginia Commonwealth University, Richmond, VA: Zhijian Chen, Mary H. Hackney and Colleen Jackson-Cook; University of Minnesota, Minneapolis, MN: Robert A. Kratzke, Diane C. Arthur and Betsy A. Hirsch; University of Puerto Rico, San Juan, Puerto Rico: Eileen I. Pacheco, Leonard L. Atkins, Cynthia C. Morton and Paola Dal Cin; University of Nebraska Medical Center, Omaha, NE: Apar Ganti and Warren G. Sanger; University of California at San Francisco, San Francisco, CA: Charalambos Andreadis and Kathleen E. Richkind; Georgetown University Medical Center, Washington, DC: Minnetta C. Liu and Jeanne M. Meck; Southern Nevada Cancer Research Foundation CCOP, Las Vegas, NV: John A. Ellerton and Marie L. Dell'Aquila.

# Methods

## Patients and treatment

A total of 1,637 patients with untreated *de novo* acute myeloid leukemia (AML) enrolled on CALGB/Alliance study protocols, detailed below, were included. CALGB is now part of the Alliance for Clinical Trials in Oncology. All patients provided written informed consent for participation in the treatment studies and in companion studies CALGB 8461 (cytogenetic studies), CALGB 9665 (leukemia tissue bank) and CALGB 20202 (molecular studies) before enrollment in accordance with the Declaration of Helsinki. Each study protocol was approved by Institutional Review Board at the respective treatment center.

For definition of race and ethnicity, the CALGB/Alliance Registration forms follow guidelines from the National Cancer Institute: 1) White: A person having origins in any of the original peoples of Europe, the Middle East, or North Africa. 2) Black or African American: A person having origins in any of the black racial groups of Africa. In our study, African-American ancestry was self-reported. 3) Hispanics or Latino refers to a person of Cuban, Mexican, Puerto Rican, South or Central American, or other Spanish culture or origin regardless of race.

Patients were treated on CALGB/Alliance studies 19808 (n=339), 10503 (n=312), 10201 (n=238), 9720 (n=229), 9621 (n=171), 9222 (n=82), 8525 (n=76), 10603 (n=70), 10502 (n=37), 8923 (n=28), 9420 (n=22), 11001 (n=10), 9022 (n=9), 8821 (n=8), 9120 (n=2), 8721 (n=2), and 10801 (n=1). Patients enrolled on CALGB 19808 were randomly assigned to receive induction chemotherapy with cytarabine, daunorubicin, and etoposide with or without PSC-833 (valspodar), a multidrug resistance protein inhibitor.^1^ On achievement of complete remission (CR), patients were assigned to intensification with high-dose cytarabine and etoposide for stem-cell mobilization followed by myeloablative treatment with busulfan and etoposide supported by autologous hematopoietic stem-cell transplantation (HSCT). Patients enrolled on CALGB 10503 were assigned to receive induction chemotherapy consisting of cytarabine, daunorubicin, and etoposide. Upon achievement of CR, patients received high-dose cytarabine (HiDAC) and etoposide for stem-cell mobilization followed by myeloablative treatment with busulfan and etoposide supported by autologous HSCT. Patients not eligible for HSCT received HiDAC. After intensification, patients received the DNA methyltransferase inhibitor decitabine for maintenance.^2^

Patients on CALGB 10201 received induction chemotherapy consisting of cytarabine and daunorubicin, with or without BCL2 antisense oblimersen sodium. The consolidation regimen included two cycles of cytarabine (2 g/m2/d) with or without oblimersen.^3^ Patients enrolled onto CALGB 9720 or 9420 received induction chemotherapy consisting of cytarabine in combination with daunorubicin and etoposide and were randomized to the arm with or without the multidrug resistance protein modulator PSC-833. The PSC-833 arm was closed after random assignment of 120 patients because of a high number of early deaths.^4-5^

Patients enrolled on CALGB 9621 were treated similarly to those on CALGB 19808, as previously reported.^6^ Patients on protocol CALGB 9222 received induction chemotherapy consisting of cytarabine in combination with daunorubicin followed by consolidation with one cycle of HiDAC. Different doses of mitoxantrone were explored as well, and the consolidation treatment was randomized to three cycles of monotherapy with HiDAC or consolidation with one cycle of HiDAC, a cycle of cyclophosphamide and etoposide, and one cycle of mitoxantrone and diaziquone.^7^ Patients enrolled onto CALGB 8525 received induction chemotherapy consisting of cytarabine in combination with daunorubicin, and were randomly assigned to consolidation with different doses of cytarabine followed by maintenance treatment.^8^ In CALGB 10603, cytarabine and daunorubicin followed by consolidation with HiDAC was applied with or without PKC-412.^9^ For patients on CALGB 10502, bortezomib was added to both induction consisting of cytarabine and daunorubicin and to consolidation with two cycles of intermediate-dose cytarabine.^10^ Patients on CALGB 8923 were treated with induction therapy consisting of cytarabine and daunorubicin and were randomly assigned to receive postremission therapy with cytarabine alone or in combination with mitoxantrone.^11^ For patients treated on CALGB 11001, sorafenib was added to the induction and consolidation treatment consisting of daunorubicin and cytarabine and consolidation with HiDAC, followed by sorafenib maintenance.^12^ Patients enrolled onto CALGB 9022 received induction chemotherapy consisting of cytarabine in combination with daunorubicin followed by consolidation with one cycle of HiDAC, a cycle of cyclophosphamide and etoposide, and one cycle of mitoxantrone and diaziquone.^13^ Patients enrolled on CALGB 8821 received cytarabine combined with daunorubicin as induction and mitoxantrone/diaziquone, and etoposide/cyclophosphamide were then successively administered in two intensification courses.^14^ Patients enrolled on CALGB 9120 received standard induction chemotherapy. After CR had been achieved, idarubicin (two days) and cytarabine (five days) were administered. Patients with histocompatible siblings were offered allogeneic HSCT, whereas the remaining patients were randomly assigned to receive a single course of high-dose cytarabine or transplantation of autologous marrow treated with perfosfamide (4-hydroperoxycyclophosphamide).^15^ Patients enrolled on CALGB 8721 received sequential treatment with HiDAC plus asparaginase. CALGB 10801 enrolled patients ≥18 years old with the favorable risk, core binding factor-positive AML. Cytarabine and daunorubicin were used for remission induction, together with other agents in some studies, and a second course of induction therapy for initial non-responders.^16^

## Cytogenetic analyses

For all CALGB/Alliance patients, pretreatment cytogenetic analyses of bone marrow (BM) and/or blood samples were performed by institutional cytogenetics laboratories using unstimulated short-term (24- and/or 48-hour) cultures, and the results were confirmed by central karyotype review as previously reported.^17^ In each patient with cytogenetically normal AML, at least 20 BM metaphase cells were analyzed and the karyotype found to be normal.

## Mutational profiling

The mutational status of the *ASXL1*, *BCOR*, *EZH2*, *NPM1*, *RUNX1*, *SF3B1*, *SRSF2*, *STAG2*, *TP53*, *U2AF1* and *ZRSR2* genes was determined by targeted amplicon sequencing using the MiSeq platform (Illumina, San Diego, CA). DNA library preparations were performed according to the manufacturer’s instructions. In brief, samples were pooled and run on the MiSeq system using the Illumina MiSeq Reagent Kit v3. Sequenced reads were aligned to the hg19 genome build using the Illumina Isis Banded Smith-Waterman aligner. Single nucleotide variant and indel calling were performed using MuTect and VarScan, respectively.^19,20^ The MuCor algorithm was used as the baseline for integrative mutation assessment.^21^ We only considered non-synonymous variants not listed in either the 1000 Genome database or dbSNP142-common variants. All called variants underwent visual inspection of the aligned reads using the Integrative Genomics Viewer (Broad Institute).^22^ All variants that occurred with VAFs of <.10 or were sequenced to a depth of <15 reads were excluded from the analysis. In addition, variants were excluded when they occurred only in one read direction if sequenced in both directions, if the region contained many variants with low quality scores, or if they occurred in all analyzed samples including run controls. In addition, samples with high background noise were entirely excluded from analysis. Samples were considered non-evaluable for a specific gene if ≥85% of the amplicons covering the target regions within the coding sequence of the gene were sequenced to a depth of <15 reads. If <15 reads were present, the gene mutation status was considered as not evaluable.

# SUPPLEMENTARY REFERENCES

1. Kolitz JE, George SL, Marcucci G, Vij R, Powell BL, Allen SL, et al. P-glycoprotein inhibition using valspodar (PSC-833) does not improve outcomes for patients under age 60 years with newly diagnosed acute myeloid leukemia: Cancer and Leukemia Group B study 19808. Blood. 2010;116:1413-21.

2. Blum W, Sanford BL, Klisovic R, DeAngelo DJ, Uy G, Powell BL, et al. Maintenance therapy with decitabine in younger adults with acute myeloid leukemia in first remission: a phase 2 Cancer and Leukemia Group B study (CALGB 10503). Leukemia. 2017;31:34-9.

3. Walker AR, Marcucci G, Yin J, Blum W, Stock W, Kohlschmidt J, et al. Phase 3 randomized trial of chemotherapy with or without oblimersen in older AML patients: CALGB 10201 (Alliance). Blood Adv. 2021;5:2775-87.

4. Baer MR, George SL, Caligiuri MA, Sanford BL, Bothun SM, Mrózek K, et al. Low-dose interleukin-2 immunotherapy does not improve outcome of patients age 60 years and older with acute myeloid leukemia in first complete remission: Cancer and Leukemia Group B study 9720. J Clin Oncol. 2008;26:4934-9.

5. Baer MR, George SL, Sanford BL, Mrózek K, Kolitz JE, Moore JO, et al. Escalation of daunorubicin and addition of etoposide.in the ADE regimen in acute myeloid leukemia patients aged 60 years and older: Cancer and Leukemia Group B Study 9720. Leukemia. 2011;25:800-7.

6. Kolitz JE, George SL, Dodge RK, Hurd DD, Powell BL, Allen SL, et al. Dose escalation studies of cytarabine, daunorubicin, and etoposide with and without multidrug resistance modulation with PSC-833 in untreated adults with acute myeloid leukemia younger than 60 years: final induction results of Cancer and Leukemia Group B study 9621. J Clin Oncol. 2004;22:4290-301.

7. Moore JO, George SL, Dodge RK, Amrein PC, Powell BL, Kolitz JE, et al. Sequential multiagent chemotherapy is not superior to high-dose cytarabine alone as postremission intensification therapy for acute myeloid leukemia in adults under 60 years of age: Cancer and Leukemia Group B study 9222. Blood. 2005;105:3420-7**.**

8. Mayer RJ, Davis RB, Schiffer CA, Berg DT, Powell BL, Schulman P, et al. Intensive postremission chemotherapy in adults with acute myeloid leukemia. N Engl J Med. 1994;331:896-903.

9. Stone RM, Mandrekar SJ, Sanford BL, Laumann K, Geyer S, Bloomfield CD, et al. Midostaurin plus chemotherapy for acute myeloid leukemia with a *FLT3* mutation. N Engl J Med. 2017;377:454-64.

10. Attar EC, Johnson JL, Amrein PC, Lozanski G, Wadleigh M, DeAngelo DJ, et al. Bortezomib added to daunorubicin and cytarabine during induction therapy and to intermediate-dose cytarabine for consolidation in patients with previously untreated acute myeloid leukemia age 60 to 75 years: CALGB (Alliance) study 10502. J Clin Oncol. 2013;31:923-9.

11. Stone RM, Berg DT, George SL, Dodge RK, Paciucci PA, Schulman P, et al. Granulocyte-macrophage colony-stimulating factor after initial chemotherapy for elderly patients with primary acute myelogenous leukemia. Cancer and Leukemia Group B. N Engl J Med. 1995;332:1671-7.

12. Uy GL, Mandrekar SJ, Laumann K, Marcucci G, Zhao W, Levis MJ, et al. A phase 2 study incorporating sorafenib into the chemotherapy for older adults with FLT3-mutated acute myeloid leukemia: CALGB 11001. Blood Adv. 2017;1:331-40.

13. Moore JO, Dodge RK, Amrein PC, Kolitz J, Lee EJ, Powell B, et al. Granulocyte-colony stimulating factor (filgrastim) accelerates granulocyte recovery after intensive postremission chemotherapy for acute myeloid leukemia with aziridinyl benzoquinone and mitoxantrone: Cancer and Leukemia Group B study 9022. Blood. 1997;89:780-8.

14. Schiffer CA, Davis RB, Schulman P, Cooper B, Coyle T, Lee E, et al. Intensive post remission therapy of acute myeloid leukemia (AML) with cytoxan/etoposide (CY/VP16) and diazaquone/mitoxantrone (AZQ/MITO). Blood. 1991;78(suppl):460 (abstract 1829).

15. Cassileth PA, Harrington DP, Appelbaum FR, Lazarus HM, Rowe JM, Paietta E, et al. Chemotherapy compared with autologous or allogeneic bone marrow transplantation in the management of acute myeloid leukemia in first remission. N Engl J Med. 1998;339:1649-1656.

16. Marcucci G, Geyer S, Laumann K, Zhao W, Bucci D, Uy GL, et al. Combination of dasatinib with chemotherapy in previously untreated core binding factor acute myeloid leukemia: CALGB 10801. Blood Adv. 2020;4:696-705.

17. Mrózek K, Carroll AJ, Maharry K, Rao KW, Patil SR, Pettenati MJ, et al. Central review of cytogenetics is necessary for cooperative group correlative and clinical studies of adult acute leukemia: the Cancer and Leukemia Group B experience. Int J Oncol. 2008;33:239-44.

18. Eisfeld AK, Mrózek K, Kohlschmidt J, Nicolet D, Orwick S, Walker CJ, et al. The mutational oncoprint of recurrent cytogenetic abnormalities in adult patients with *de novo* acute myeloid leukemia. Leukemia. 2017;31:2211-8.

19. Cibulskis K, Lawrence MS, Carter SL, Sivachenko A, Jaffe D, Sougnez C, et al. Sensitive detection of somatic point mutations in impure and heterogeneous cancer samples. Nat Biotechnol. 2013;31:213-9.

20. DePristo MA, Banks E, Poplin R, Garimella KV, Maguire JR, Hartl C, et al. A framework for variation discovery and genotyping using next-generation DNA sequencing data. Nat Genet. 2011;43:491-8.

21. Kroll KW, Eisfeld A-K, Lozanski G, Bloomfield CD, Byrd JC, Blachly JS. MuCor: mutation aggregation and correlation. Bioinformatics. 2016;32:1557-8.

22. Robinson JT, Thorvaldsdóttir H, Winckler W, Guttman M, Lander ES, Getz G, et al. Integrative genomics viewer. Nat Biotechnol. 2011;29:24-6.

# Supplementary Table 1. Multivariable analyses of outcome in patients with de novo AML categorized according to the 2022 ELN genetic-risk classification

| **Variable in final model^a^** | **Complete remission rate** | | **Disease-free survival** | | **Overall survival** | |
| --- | --- | --- | --- | --- | --- | --- |
|  | **OR (95% CI)** | ***P*** | **HR (95% CI)** | ***P*** | **HR (95% CI)** | ***P*** |
| 2022 ELN genetic-risk group^b^  Favorable vs adverse  Intermediate vs adverse | 5.64 (4.26-7.48)  2.38 (1.78-3.17) | <0.001 | 0.41 (0.35-0.49)  0.73 (0.60-0.88) | <0.001 | 0.35 (0.31-0.41)  0.67 (0.58-0.78) | <0.001 |
| Hemoglobin (continuous, each 1 unit increase) | 1.10 (1.03-1.17) | 0.001 | — | — | — | — |
| WBC (continuous, each 50 unit increase) | 0.81 (0.73-0.90) | <0.001 | 1.11 (1.04-1.18) | 0.002 | 1.11 (1.07-1.16) | <0.001 |
| Age (continuous, each 10 unit increase) | 0.734 (0.69-0.80) | <0.001 | 1.20 (1.14-1.26) | <0.001 | 1.33 (1.28-1.38) | <0.001 |
| Platelets (continuous, each 50 unit increase) | — | — | 0.92 (0.87-0.98) | 0.006 | 0.95 (0.91-0.99) | 0.009 |
| Years of diagnosis  1986-1994 vs 2005-2013  1995-2004 vs 2005-2013 | — | — | 1.49 (1.17-1.90)  1.33 (1.14-1.56) | <0.001 | 1.42 (1.17-1.71)  1.23 (1.10-1.40) | <0.001 |

Odds ratios greater than (less than) 1.0 mean higher (lower) complete remission rate for the higher values of the continuous variables and the first category listed for the categorical variables. Hazard ratios greater than (less than) 1.0 indicate higher (lower) risk for relapse or death (disease-free survival) or death (overall survival) for the higher values of the continuous variables and the first category listed for the categorical variables. Variables considered in the model were those significant at α=.20 from the univariable models.

Abbreviations: OR, odds ratio; HR, hazard ratio; CI, confidence interval; ELN, European LeukemiaNet; WBC, white blood cell count.

^a^ Variables considered for all outcome endpoints were as follows: 2022 ELN risk-groups, age (in 10-year increments), sex (male vs female), WBC count (in 50-unit increments), platelets (in 50-unit increments), hemoglobin (in 1-unit increments), years of diagnosis (1986-1994 vs 2005-2013 and 1995-2004 vs 2005-2013), and extramedullary involvement (present v absent).

^b^ The 2022 ELN classification was considered as a three-level categorical variable, for which the ELN adverse group was used as a reference group.

**Supplementary Table 2.** Treatment outcome of younger patients (aged <60 years) with AML categorized according to the 2022 ELN genetic-risk classification

#

| **Endpoint** | **Favorable**  **n=439**  **(I)** | **Intermediate**  **n=278**  **(II)** | **Adverse**  **n=323**  **(III)** | ***P*^a^**  **I vs II** | ***P*^a^**  **I vs III** | ***P*^a^**  **II vs III** |
| --- | --- | --- | --- | --- | --- | --- |
| Complete remission rate, n (%)^b^ | 388 (88) | 204 (73) | 171 (53) | <0.001 | <0.001 | <0.001 |
| Relapse rate, n (%) | 176 (46) | 115 (67) | 113 (86) | <0.001 | <0.001 | <0.001 |
| Disease-free survival  Median, years  % Disease-free at 1 year (95% CI)  % Disease-free at 3 years (95% CI)  % Disease-free at 5 years (95% CI) | 4.5  70 (65-74) 53 (47-57) 49 (44-54) | 0.8  45 (38-53) 27 (21-34) 27 (20-33) | 0.7 36 (28-44) 12 (7-18) 7 (4-13) | <0.001 | <0.001 | <0.001 |
| Overall survival  Median, years  % Alive at 1 year (95% CI)  % Alive at 3 years (95% CI)  % Alive at 5 years (95% CI) | 11.6  83 (79-86) 62 (57-67) 56 (51-61) | 1.2  57 (51-63) 33 (27-39) 29 (24-35) | 0.9 44 (38-50) 16 (12-21) 12 (9-16) | <0.001 | <0.001 | <0.001 |

^a^ *P*-values are from the Fisher’s exact test for complete remission (CR) and relapse and the log rank test for disease-free and overall survival and are for the specified two-way comparisons.

^b^ For CR analyses the denominator included patients who received an allogeneic hematopoietic stem-cell transplantation in first CR. Relapse rate, disease-free and overall survival analyses exclude patients who received an allogeneic hematopoietic stem-cell transplantation in first CR (favorable: n = 430, those who achieved a CR n = 379; intermediate: n = 246, those who achieved a CR n = 172; adverse: n = 283, those who achieved a CR n = 132).

# Supplementary Table 3. Treatment outcome of older patients (aged ≥60 years) with AML categorized according to the 2022 ELN genetic-risk classification

| **Endpoint** | **Favorable**  **n=141**  **(I)** | **Intermediate**  **n=112**  **(II)** | **Adverse**  **n=344**  **(III)** | ***P*^a^**  **I vs II** | ***P*^a^**  **I vs III** | ***P*^a^**  **II vs III** |
| --- | --- | --- | --- | --- | --- | --- |
| Complete remission rate, n (%)^b^ | 97 (69) | 60 (54) | 120 (35) | 0.02 | <0.001 | <0.001 |
| Relapse rate, n (%) | 69 (78) | 51 (89) | 96 (88) | 0.08 | 0.06 | 1.00 |
| Disease-free survival  Median, years  % Disease-free at 1 year (95% CI)  % Disease-free at 3 years (95% CI)  % Disease-free at 5 years (95% CI) | 1.0  52 (41-61) 29 (20-39)  22 (14-32) | 0.5  16 (8-26)  7 (2-16)   5 (1-13) | 0.5 25 (17-33) 6 (2-11) 5 (2-10) | <0.001 | <0.001 | 0.27 |
| Overall survival  Median, years  % Alive at 1 year (95% CI)  % Alive at 3 years (95% CI)  % Alive at 5 years (95% CI) | 1.3  58 (49-65) 27 (19-34) 20 (14-28) | 0.6  23 (16-31) 9 (5-15) 6 (3-12) | 0.4 21 (17-26) 5 (3-8) 2 (1-4) | <0.001 | <0.001 | 0.09 |

^a^ *P*-values are from the Fisher’s exact test for complete remission (CR) and relapse and the log rank test for disease-free and overall survival and are for the specified two-way comparisons.

^b^ For CR analyses the denominator included patients who received an allogeneic hematopoietic stem-cell transplantation in first CR. Relapse rate, disease-free and overall survival analyses exclude patients who received an allogeneic hematopoietic stem-cell transplantation in first CR (favorable: n = 132, those who achieved a CR n = 89; intermediate: n = 109, those who achieved a CR n = 57; adverse: n = 333, those who achieved a CR n = 108).

# Supplementary Table 4. Treatment outcome of younger (aged <60 years) patients of African American ancestry with AML categorized according to the 2022 ELN genetic-risk classification

| **Endpoint** | **Favorable**  **n=29**  **(I)** | **Intermediate**  **n=19**  **(II)** | **Adverse**  **n=29**  **(III)** | ***P*^a^**  **I vs II** | ***P*^a^**  **I vs III** | ***P*^a^**  **II vs III** |
| --- | --- | --- | --- | --- | --- | --- |
| Complete remission rate, n (%)^b^ | 24 (83) | 12 (63) | 19 (66) | 0.18 | 0.23 | 1.00 |
| Relapse rate, n (%) | 14 (58) | 5 (50) | 19 (100) | 0.72 | 0.001 | 0.002 |
| Disease-free survival  Median, years  % Disease-free at 1 year (95% CI)  % Disease-free at 3 years (95% CI)  % Disease-free at 5 years (95% CI) | 1.7  58 (36-75) 42 (22-60) 32 (15-51) | 0.6  30 (7-58) 30 (7-58) 30 (7-58) | 0.7  32 (13-52)  0   0 | 0.42 | 0.002 | 0.30 |
| Overall survival  Median, years  % Alive at 1 year (95% CI)  % Alive at 3 years (95% CI)  % Alive at 5 years (95% CI) | 2.6  79 (60-90) 48 (29-65) 45 (27-62) | 0.8  41 (19-63) 24 (7-45)  24 (7-45) | 0.9  48 (29-65) 4 (4-29)  3 (0-15) | 0.05 | <0.001 | 0.46 |

^a^ *P*-values are from the Fisher’s exact test for complete remission (CR) and relapse and the log rank test for disease-free and overall survival and are for the specified two-way comparisons.

^b^ For CR analyses the denominator included patients who received an allogeneic hematopoietic stem-cell transplantation in first CR. Relapse rate, disease-free and overall survival analyses exclude patients who received an allogeneic hematopoietic stem-cell transplantation in first CR (favorable: n = 29, those who achieved a CR n = 24; intermediate: n = 17, those who achieved a CR n = 10; adverse: n = 29, those who achieved a CR n = 19).

# Supplementary Table 5. Treatment outcome of younger (aged <60 years) Hispanic patients with AML categorized according to the 2022 ELN genetic-risk classification

| **Endpoint** | **Favorable**  **n=18**  **(I)** | **Intermediate**  **n=7**  **(II)** | **Adverse**  **n=12**  **(III)** | ***P*^a^**  **I vs II** | ***P*^a^**  **I vs III** | ***P*^a^**  **II vs III** |
| --- | --- | --- | --- | --- | --- | --- |
| Complete remission rate, n (%) | 15 (83) | 6 (86) | 6 (50) | 1.00 | 0.10 | 0.17 |
| Relapse rate, n (%) | 7 (47) | 2 (33) | 5 (83) | 0.66 | 0.18 | 0.24 |
| Disease-free survival  Median, years  % Disease-free at 1 year (95% CI)  % Disease-free at 3 years (95% CI)  % Disease-free at 5 years (95% CI) | 2.4  60 (32-80) 47 (21-69) 47 (21-69) | 10.8  67 (19-90) 67 (19-90) 67 (19-90) | 0.4  17 (1-52) 17 (1-52) 17 (1-52) | 0.42 | 0.04 | 0.02 |
| Overall survival  Median, years  % Alive at 1 year (95% CI)  % Alive at 3 years (95% CI)  % Alive at 5 years (95% CI) | 10.5  78 (51-91) 67 (40-83) 61 (35-79) | 10.9  86 (33-98) 71 (26-92) 71 (26-92) | 0.6  33 (10-59)  8 (1-31)  8 (1-31) | 0.67 | 0.004 | 0.01 |

^a^ *P*-values are from the Fisher’s exact test for complete remission and relapse and the log rank test for disease-free and overall survival and are for the specified two-way comparisons.

# Supplementary Table 6. Treatment outcomes of patients with AML and myelodysplasia-related mutations and no favorable genetic features, of patients with adverse-risk markers other than myelodysplasia-related mutations, and of patients with myelodysplasia-related mutations co-occurring with favorable-risk AML subtypes

| **Endpoint** | **Adverse myelodysplasia-related**  **n=360**  **(I)** | **Other adverse**  **n=307**  **(II)** | **Favorable**  **myelodysplasia-related**  **n=96**  **(III)** | ***P*^a^**  **I vs II** | ***P*^a^**  **I vs III** |
| --- | --- | --- | --- | --- | --- |
| Complete remission rate, n (%)^b^ | 155 (43) | 136 (44) | 70 (73) | 0.75 | <0.001 |
| Relapse rate, n (%) | 116 (88) | 93 (85) | 42 (61) | 0.57 | <0.001 |
| Disease-free survival  Median, years  % Disease-free at 1 year (95% CI)  % Disease-free at 3 years (95% CI)  % Disease-free at 5 years (95% CI) | 0.7   36 (28-44)   7 (3-12)   5 (2-9) | 0.5   25 (17-33)   12 (7-19)   8 (4-14) | 1.9   61 (48-71)   42 (30-53)   39 (28-50) | 0.10 | <0.001 |
| Overall survival  Median, years  % Alive at 1 year (95% CI)  % Alive at 3 years (95% CI)  % Alive at 5 years (95% CI) | 0.7   38 (33-43)   12 (9-16)   7 (5-10) | 0.5   24 (19-29)   9 (6-12)   7 (4-10) | 2.0   68 (58-77)   46 (36-56)   39 (29-48) | 0.005 | <0.001 |

^a^ *P*-values are from the Fisher’s exact test for complete remission (CR) and relapse and the log rank test for disease-free and overall survival and are for the specified two-way comparisons.

^b^ For CR analyses the denominator included patients who received an allogeneic hematopoietic stem-cell transplantation in first CR. Relapse rate, disease-free and overall survival analyses exclude patients who received an allogeneic hematopoietic stem-cell transplantation in first CR (adverse myelodysplasia-related: n = 337, those who achieved a CR n = 131; other adverse: n = 279 those who achieved a CR n = 109; favorable myelodysplasia-related: n = 95, those who achieved a CR n = 69).

# Supplementary Table 7. Outcomes of patients with myelodysplasia-related mutations co-occurring with favorable-risk AML subtypes versus patients with other favorable-risk markers

| **Endpoint** | **Favorable with**  **myelodysplasia-related mutations**  **n=96** | **Other**  **Favorable**  **n=484** | ***P*^a^** |
| --- | --- | --- | --- |
| Complete remission rate, n (%)^b^ | 70 (73) | 415 (86) | 0.004 |
| Relapse rate, n (%) | 42 (61) | 203 (51) | 0.15 |
| Disease-free survival  Median, years  % Disease-free at 1 year (95% CI)  % Disease-free at 3 years (95% CI)  % Disease-free at 5 years (95% CI) | 1.9   61 (48-71)   42 (30-53)   39 (28-50) | 2.8   68 (63-72)   49 (44-54)   45 (40-49) | 0.14 |
| Overall survival  Median, years  % Alive at 1 year (95% CI)  % Alive at 3 years (95% CI)  % Alive at 5 years (95% CI) | 2.0   68 (58-77)   46 (36-56)   39 (29-48) | 4.8   79 (75-82)   55 (51-60)   50 (45-54) | 0.003 |

^a^ *P*-values are from the Fisher’s exact test for complete remission (CR) and relapse and the log rank test for disease-free and overall survival.

^b^ For CR analyses the denominator included patients who received an allogeneic hematopoietic stem-cell transplantation in first CR. Relapse rate, disease-free and overall survival analyses exclude patients who received an allogeneic hematopoietic stem-cell transplantation in first CR (favorable with myelodysplasia-related mutations: n = 467, those who achieved a CR n = 399; other favorable: n = 95, those who achieved a CR n = 69).

# Supplementary Table 8. Outcomes of ELN 2022 favorable patients with core-binding (CBF-AML) and myelodysplasia-related mutations versus the remaining patients with CBF-AML

| **Endpoint** | **CBF-AML with**  **myelodysplasia-related mutations**  **n=15** | **Other CBF-AML**  **n=158** | ***P*^a^** |
| --- | --- | --- | --- |
| Complete remission rate, n (%)^b^ | 14 (93) | 146 (92) | 1.00 |
| Relapse rate, n (%) | 6 (43) | 59 (43) | 1.00 |
| Disease-free survival  Median, years  % Disease-free at 1 year (95% CI)  % Disease-free at 3 years (95% CI)  % Disease-free at 5 years (95% CI) | NR   57 (28-78)   57 (28-78)   57 (28-78) | 6.9   69 (61-76)   54 (46-62)   51 (43-59) | 0.65 |
| Overall survival  Median, years  % Alive at 1 year (95% CI)  % Alive at 3 years (95% CI)  % Alive at 5 years (95% CI) | NR   93 (61-99)   73 (44-89)   66 (36-84) | 13.3   88 (81-92)   66 (58-73)   63 (55-70) | 0.65 |

NR, not reached.

^a^ *P*-values are from the Fisher’s exact test for complete remission (CR) and relapse and the log rank test for disease-free and overall survival.

^b^ For CR analyses the denominator included patients who received an allogeneic hematopoietic stem-cell transplantation in first CR. Relapse rate, disease-free and overall survival analyses exclude patients who received an allogeneic hematopoietic stem-cell transplantation in first CR (CBF-AML with myelodysplasia-related mutations: n = 15, those who achieved a CR n = 14; other CBF-AML: n = 147, those who achieved a CR n = 136).

# Supplementary Table 9. Outcomes of ELN 2022 favorable patients with *CEBPA*^bZip^ and myelodysplasia-related mutations and of other patients with *CEBPA*^bZip^ mutations

| **Endpoint** | ***CEBPA*^bZip^ and**  **myelodysplasia-related mutations n=21** | **Other *CEBPA*^bZip^**  **mutations**  **n=82** | ***P*^a^** |
| --- | --- | --- | --- |
| Complete remission rate, n (%) | 16 (76) | 70 (85) | 0.33 |
| Relapse rate, n (%) | 10 (63) | 37 (53) | 0.58 |
| Disease-free survival  Median, years  % Disease-free at 1 year (95% CI)  % Disease-free at 3 years (95% CI)  % Disease-free at 5 years (95% CI) | 1.6   63 (35-81)   38 (15-60)   38 (15-60) | 3.0   66 (53-76)   49 (36-60)   43 (31-54) | 0.74 |
| Overall survival  Median, years  % Alive at 1 year (95% CI)  % Alive at 3 years (95% CI)  % Alive at 5 years (95% CI) | 2.3   76 (52-89)   43 (22-62)   38 (18-58) | 4.8   83 (73-90)   59 (47-68)   50 (39-60) | 0.49 |

**^a^** *P*-values are from the Fisher’s exact test for complete remission and relapse and the log rank test for disease-free and overall survival.

# Supplementary Table 10. Outcomes of ELN 2022 favorable patients with *NPM1* and myelodysplasia-related mutations without *FLT3*-ITD versus other *NPM1*-mutated patients without *FLT3*-ITD

| **Endpoint** | ***NPM1*-mutated /*FLT3*-ITD‒ and**  **myelodysplasia-** **related mutations**  **n=67** | **Other *NPM1*-mutated/**  ***FLT3*-ITD‒**  **n=258** | ***P*^a^** |
| --- | --- | --- | --- |
| Complete remission rate, n (%)^b^ | 45 (67) | 210 (81) | 0.02 |
| Relapse rate, n (%) | 30 (70) | 111 (54) | 0.09 |
| Disease-free survival  Median, years  % Disease-free at 1 year (95% CI)  % Disease-free at 3 years (95% CI)  % Disease-free at 5 years (95% CI) | 1.3   63 (47-75)   35 (21-49)   30 (17-44) | 2.4   67 (60-73)   47 (40-53)   43 (36-49) | 0.03 |
| Overall survival  Median, years  % Alive at 1 year (95% CI)  % Alive at 3 years (95% CI)  % Alive at 5 years (95% CI) | 1.5   62 (49-72)   40 (28-52)   32 (21-44) | 3.0   73 (67-78)   49 (43-55)   42 (36-48) | 0.005 |

^a^ *P*-values are from the Fisher’s exact test for complete remission (CR) and relapse and the log rank test for disease-free and overall survival.

^b^ For CR analyses the denominator included patients who received an allogeneic hematopoietic stem-cell transplantation in first CR. Relapse rate, disease-free and overall survival analyses exclude patients who received an allogeneic hematopoietic stem-cell transplantation in first CR (*NPM1*-mutated/*FLT3*-ITD-negative with myelodysplasia-related mutations: n = 65, those who achieved a CR n = 43; other *NPM1*-mutated/*FLT3*-ITD-negative: n = 252, those who achieved a CR n = 204).

# Supplementary Table 11. Outcome of *NPM1*-mutated patients without *FLT3*-ITD with myelodysplasia-related mutations and patients in the 2022 ELN intermediate group

| **Endpoint** | ***NPM1*-mutated /*FLT3*-ITD‒ &**  **myelodysplasia-** **related mutations**  **n=67** | **Intermediate**  **n=390** | ***P*^a^** |
| --- | --- | --- | --- |
| Complete remission rate, n (%)^b^ | 45 (67) | 264 (68) | 1.00 |
| Relapse rate, n (%) | 30 (70) | 166 (72) | 0.71 |
| Disease-free survival  Median, years  % Disease-free at 1 year (95% CI)  % Disease-free at 3 years (95% CI)  % Disease-free at 5 years (95% CI) | 1.3   63 (47-75)   35 (21-49)   30 (17-44) | 0.7   38 (32-44)   22 (17-28)   21 (16-27) | 0.19 |
| Overall survival  Median, years  % Alive at 1 year (95% CI)  % Alive at 3 years (95% CI)  % Alive at 5 years (95% CI) | 1.5   62 (49-72)   40 (28-52)   32 (21-44) | 0.9   47 (41-52)   26 (21-30)   22 (18-27) | 0.28 |

^a^ *P*-values are from the Fisher’s exact test for complete remission (CR) and relapse and the log rank test for disease-free and overall survival.

^b^ For CR analyses the denominator included patients who received an allogeneic hematopoietic stem-cell transplantation in first CR. Relapse rate, disease-free and overall survival analyses exclude patients who received an allogeneic hematopoietic stem-cell transplantation in first CR (*NPM1*-mutated/*FLT3*-ITD-negative with myelodysplasia-related mutations: n = 65, those who achieved a CR n = 43; intermediate: n = 355, those who achieved a CR n = 229).

# Supplementary Table 12. Outcome of *NPM1*-mutated/*FLT3*-ITD-negative patients, of those with adverse cytogenetics, and patients in the 2022 ELN adverse group

| **Endpoint** | ***NPM1*-mutated/ *FLT3*-ITD‒**  **n=305**  **(I)** | ***NPM1*-mutated/ *FLT3*-ITD‒ with adverse cytogenetics**  **n=14**  **(II)** | **Adverse**  **n=653**  **(III)** | ***P*^a^**  **I vs II** | ***P*^a^**  **II vs III** |
| --- | --- | --- | --- | --- | --- |
| Complete remission rate, n (%)^b^ | 240 (79) | 9 (64) | 282 (43) | 0.20 | 0.17 |
| Relapse rate, n (%) | 130 (56) | 7 (88) | 202 (87) | 0.14 | 1.00 |
| Disease-free survival  Median, years  % Disease-free at 1 year (95% CI)  % Disease-free at 3 years (95% CI)  % Disease-free at 5 years (95% CI) | 2.4   68 (61-73)   45 (39-51)   41 (34-47) | 1.1  50 (15-77)  38 (9-67)  38 (9-67) | 0.6   30 (24-36)   8 (5-12)   5 (3-8) | 0.06 | 0.12 |
| Overall survival  Median, years  % Alive at 1 year (95% CI)  % Alive at 3 years (95% CI)  % Alive at 5 years (95% CI) | 2.7   71 (65-76)   47 (42-53)   41 (35-46) | 1.0  54 (25-76)  31 (9-55)  23 (6-47) | 0.6   31 (27-35)   10 (8-13)   7 (5-9) | 0.04 | 0.08 |

**^a^** *P*-values are from the Fisher’s exact test for complete remission (CR) and relapse and the log rank test for disease-free and overall survival and are for the specified two-way comparisons.

^b^ For CR analyses the denominator included patients who received an allogeneic hematopoietic stem-cell transplantation in first CR. Relapse rate, disease-free and overall survival analyses exclude patients who received an allogeneic hematopoietic stem-cell transplantation in first CR (*NPM1*-mutated/*FLT3*-ITD-negative: n = 298, those who achieved a CR n = 233; *NPM1*-mutated/*FLT3*-ITD-negative with adverse cytogenetics: n = 13. those who achieved a CR n = 8; other adverse: n = 603, those who achieved a CR n = 232).

# Supplementary Table 13. Outcome of *NPM1*-mutated/*FLT3*-ITD-negative patients with adverse cytogenetics and patients in the 2022 ELN intermediate group

| **Endpoint** | ***NPM1*-mutated/ *FLT3*-ITD‒ with adverse cytogenetics**  **n=14** | **Intermediate**  **n=390** | ***P*^a^** |
| --- | --- | --- | --- |
| Complete remission rate, n (%)^b^ | 9 (64) | 264 (68) | 0.78 |
| Relapse rate, n (%) | 7 (88) | 166 (72) | 0.69 |
| Disease-free survival  Median, years  % Disease-free at 1 year (95% CI)  % Disease-free at 3 years (95% CI)  % Disease-free at 5 years (95% CI) | 1.1  50 (15-77)  38 (9-67)  38 (9-67) | 0.7   38 (32-44)   22 (17-28)   21 (16-27) | 0.94 |
| Overall survival  Median, years  % Alive at 1 year (95% CI)  % Alive at 3 years (95% CI)  % Alive at 5 years (95% CI) | 1.0  54 (25-76)  31 (9-55)  23 (6-47) | 0.9   47 (41-52)   26 (21-30)   22 (18-27) | 0.72 |

^a^ *P*-values are from the Fisher’s exact test for complete remission (CR) and relapse and the log rank test for disease-free and overall survival.

^b^ For CR analyses the denominator included patients who received an allogeneic hematopoietic stem-cell transplantation in first CR. Relapse rate, disease-free and overall survival analyses exclude patients who received an allogeneic hematopoietic stem-cell transplantation in first CR (*NPM1*-mutated/*FLT3*-ITD-negative with adverse cytogenetics: n = 13, those who achieved a CR n = 8; intermediate: n = 355, those who achieved a CR n = 229).

# Supplementary Table 14. Outcome of patients with *FLT3*-ITD classified in the intermediate risk-group versus other patients in the intermediate risk-group versus patients in the adverse risk-group^a^

| **Endpoint** | **Intermediate**  **with *FLT3*-ITD**  **n=188**  **(I)** | **Other intermediate**  **n=157**  **(II)** | **Adverse**  **n=658**  **(III)** | ***P***^b^  **I vs II** | ***P***^b^  **I vs III** |
| --- | --- | --- | --- | --- | --- |
| Complete remission rate, n (%)^c^ | 119 (63) | 114 (73) | 287 (43) | 0.08 | <0.001 |
| Relapse rate, n (%) | 93 (81) | 60 (65) | 207 (87) | 0.01 | 0.21 |
| Disease-free survival  Median, years  % Disease-free at 1 year (95% CI)  % Disease-free at 3 years (95% CI)  % Disease-free at 5 years (95% CI) | 0.6  27 (19-35)  16 (10-23)  16 (10-23 | 1.0   49 (39-59)   30 (21-40)   28 (19-37) | 0.6  31 (25-37)  9 (6-13)  6 (4-10 | <0.001 | 0.34 |
| Overall survival  Median, years  % Alive at 1 year (95% CI)  % Alive at 3 years (95% CI)  % Alive at 5 years (95% CI) | 0.7  34 (27-41)  19 (14-25)  17 (12-23) | 1.3   60 (52-68)   33 (25-41)   28 (21-36) | 0.6  31 (28-35)  10 (8-13)  7 (5-9) | <0.001 | <0.001 |

^a^ Patients who received treatment with midostaurin were excluded from the analyses.

^b^ *P*-values are from the Fisher’s exact test for complete remission (CR) and relapse and the log rank test for disease-free and overall survival and are for the specified two-way comparisons.

^c^ For CR analyses the denominator included patients who received an allogeneic hematopoietic stem-cell transplantation in first CR. Relapse rate, disease-free and overall survival analyses exclude patients who received an allogeneic hematopoietic stem-cell transplantation in first CR (intermediate with *FLT3*-ITD: n = 184, those who achieved a CR n = 115; other intermediate: n = 136, those who achieved a CR n = 93; adverse: n = 609, those who achieved a CR n = 238).

# Supplementary Table 15. Outcome of patients with *FLT3*-ITD classified in the intermediate risk-group who received treatment with midostaurin versus those who did not

| **Endpoint** | **Intermediate**  **with *FLT3*-ITD receiving midostaurin**  **n=22** | **Intermediate**  **with *FLT3*-ITD**  **receiving only chemotherapy**  **n=208** | ***P*^a^** |
| --- | --- | --- | --- |
| Complete remission rate, n (%)^b^ | 15 (65) | 133 (64) | 0.82 |
| Relapse rate, n (%) | 7 (78) | 99 (79) | 1.00 |
| Disease-free survival  Median, years  % Disease-free at 1 year (95% CI)  % Disease-free at 3 years (95% CI)  % Disease-free at 5 years (95% CI) | 1.2   67 (28-88)   22 (3-51)   22 (3-51) | 0.6   27 (20-35)   16 (10-23)   16 (10-23) | 0.14 |
| Overall survival  Median, years  % Alive at 1 year (95% CI)  % Alive at 3 years (95% CI)  % Alive at 5 years (95% CI) | 1.5   75 (46-90)   31 (11-54)   31 (11-54) | 0.7   35 (28-42)   20 (14-25)   17 (13-23) | 0.11 |

**^a^** *P*-values are from the Fisher’s exact test for complete remission (CR) and relapse and the log rank test for disease-free and overall survival.

^b^ For CR analyses the denominator included patients who received an allogeneic hematopoietic stem-cell transplantation in first CR. Relapse rate, disease-free and overall survival analyses exclude patients who received an allogeneic hematopoietic stem-cell transplantation in first CR (intermediate with *FLT3*-ITD receiving midostaurin: n = 16, those who achieved a CR n = 9; Intermediate with *FLT3*-ITD no midostaurin: n = 201, those who achieved a CR n = 126).

# Supplementary Table 16. Outcomes of patients with *CEBPA*^bZIP^ vs *CEBPA*^bZIP^ with bi-allelic *CEBPA* vs bi-allelic *CEBPA*

| **Endpoint** | **Monoallelic *CEBPA*^bZip^**  **mutations**  **n=39**  **(I)** | **Biallelic *CEBPA*^bZip^**  **mutations**  **n=64**  **(II)** | **Biallelic other *CEBPA***  **mutations**  **n=31**  **(III)** | ***P*^a^**  **I vs II** | ***P*^a^**  **I vs III** | ***P*^a^**  **II vs III** |
| --- | --- | --- | --- | --- | --- | --- |
| Complete remission rate, n (%)^b^ | 25 (64) | 61 (95) | 20 (65) | <0.001 | 1.00 | <0.001 |
| Relapse rate, n (%) | 16 (64) | 31 (51) | 8 (47) | 0.34 | 0.35 | 1.00 |
| Disease-free survival  Median, years  % Disease-free at 1 year (95% CI)  % Disease-free at 3 years (95% CI)  % Disease-free at 5 years (95% CI) | 1.4   64 (42-79)   40 (21-58)   36 (18-54) | 3.0   66 (52-76)   49 (36-61)   44 (31-56) | 0.5  35 (14-57)  35 (14-57)  35 (14-57 | 0.27 | 0.51 | 0.12 |
| Overall survival  Median, years  % Alive at 1 year (95% CI)  % Alive at 3 years (95% CI)  % Alive at 5 years (95% CI) | 2.0   67 (50-79)   44 (28-58)   38 (24-53) | 6.5   91 (80-96)   63 (49-73)   53 (40-64) | 0.7  36 (19-53)  21 (9-38)  21 (9-38) | 0.009 | 0.07 | <0.001 |

**^a^** *P*-values are from the Fisher’s exact test for complete remission (CR) and relapse and the log rank test for disease-free and overall survival and are for the specified two-way comparisons.

^b^ For CR analyses the denominator included patients who received an allogeneic hematopoietic stem-cell transplantation in first CR. Relapse rate, disease-free and overall survival analyses exclude patients who received an allogeneic hematopoietic stem-cell transplantation in first CR (monoallelic *CEBPA*^bZip^  mutations: n = 39, those who achieved a CR n = 25; biallelic *CEBPA*^bZip^ mutations: n = 64, those who achieved a CR n = 61; biallelic other CEBPA mutations: n = 28, those who achieved a CR n = 17).

**Supplementary Figure 1**. **Comparison of risk group assignments between the 2017 and 2022 ELN genetic-risk classifications showing reallocation of patients with AML from 2017 into 2022 ELN categories. a** The entire patient cohort, **b** younger adults under the age of 60 years and **c** older patients aged 60 years or older.

**a**

**
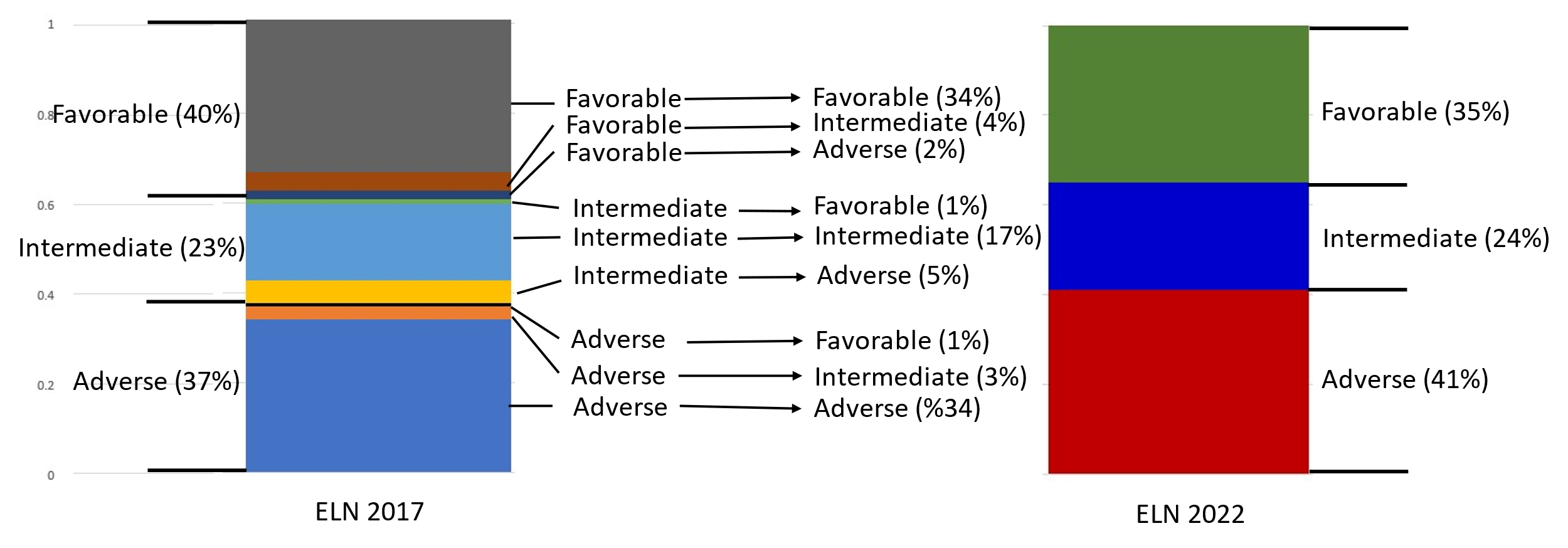
**

**b**

**
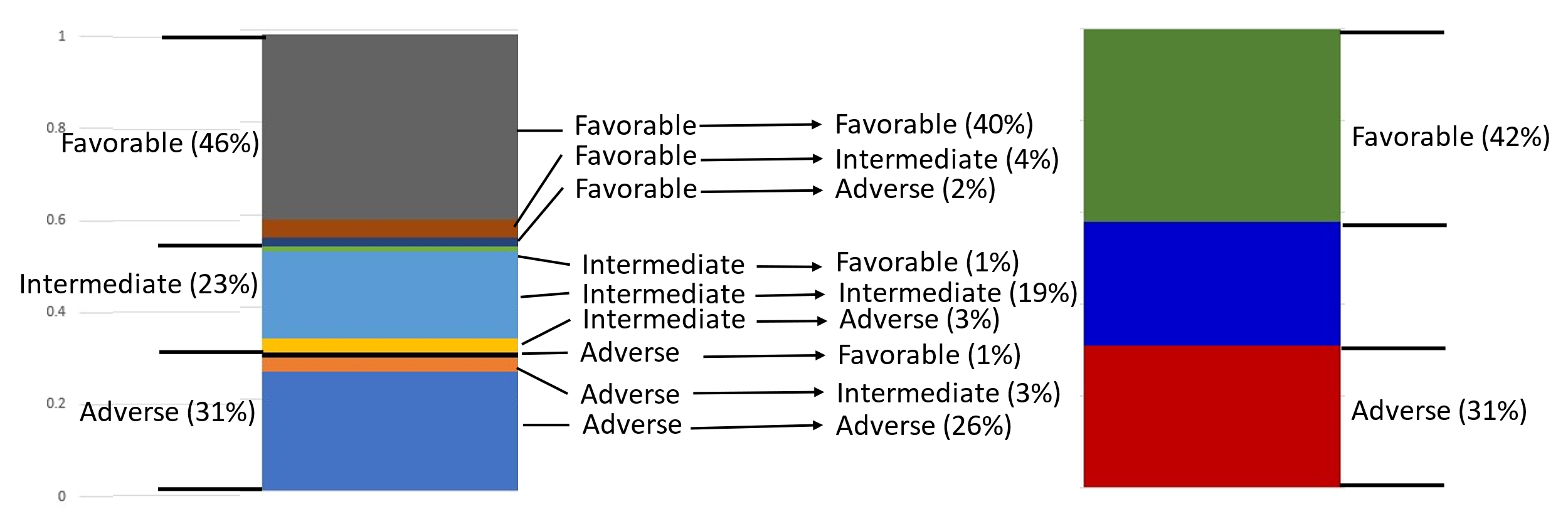
**

**c**

**
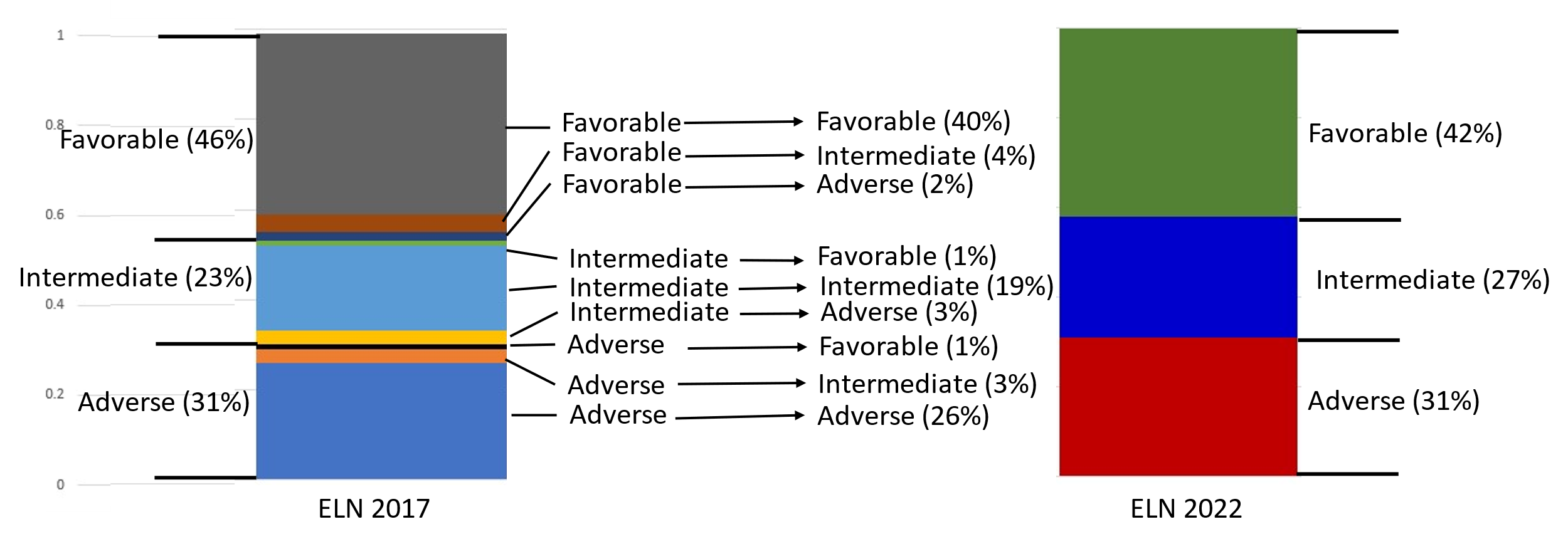
**

**Supplementary** **Figure 2. The receiver operating characteristic (ROC) curves illustrating the ability of 2022 European LeukemiaNet (ELN) genetic‑risk classification and 2017 ELN genetic‑risk classification to predict outcome in a whole cohort of patients with AML. a** Achievement of complete remission, **b** relapse rates, **c** 3‑year disease-free survival rates and **d** 3‑year overall survival rates.

# a b

#
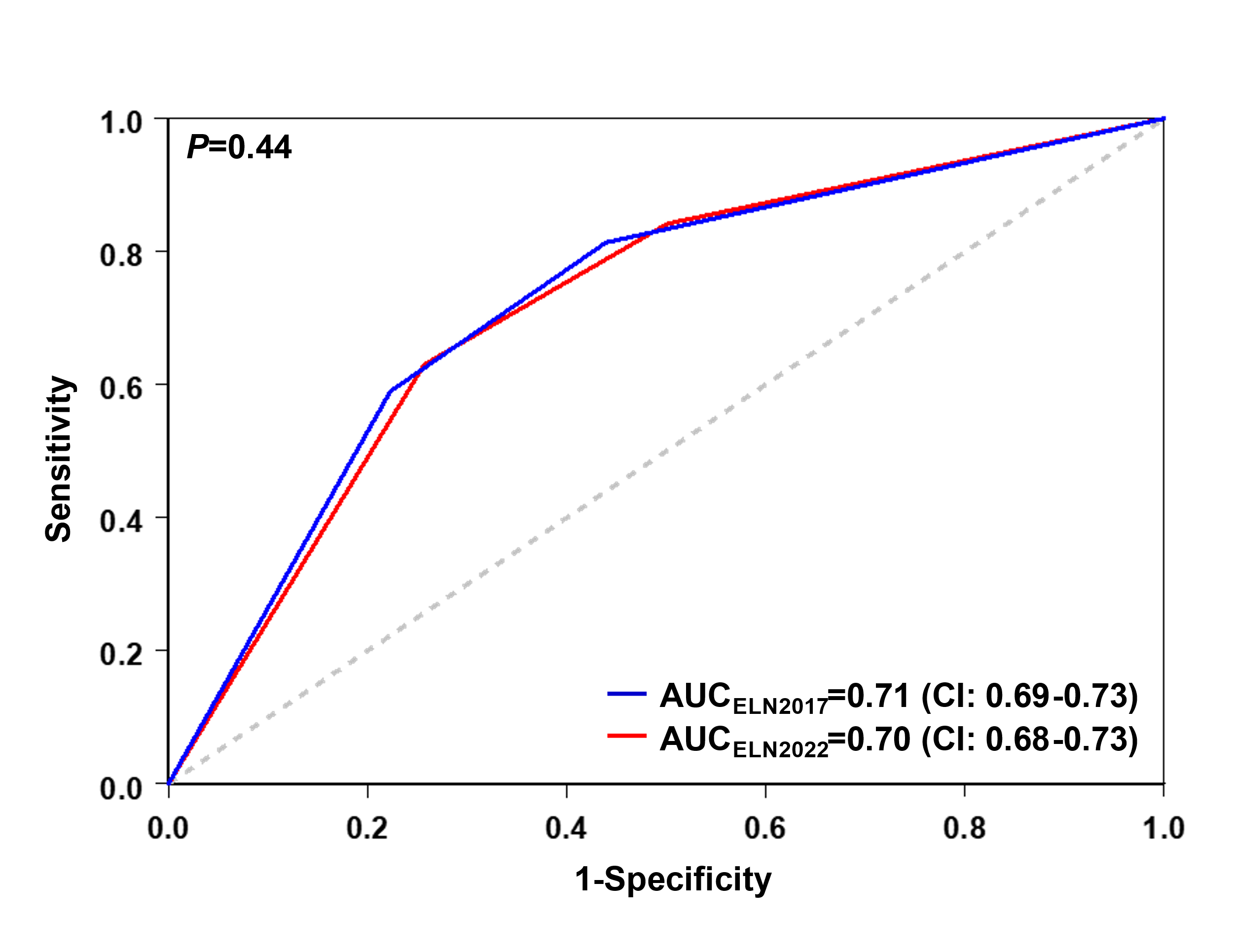

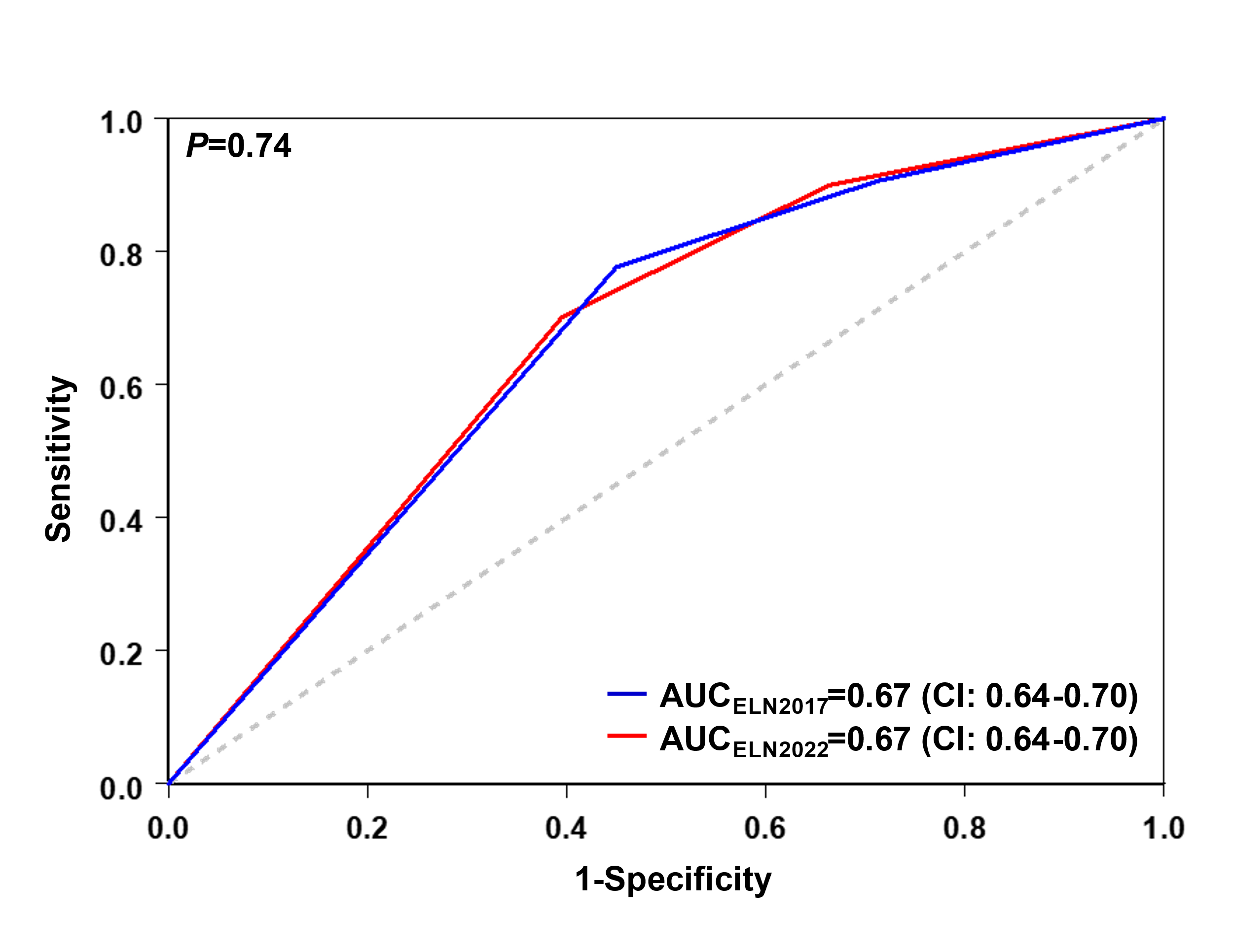


# c d

#
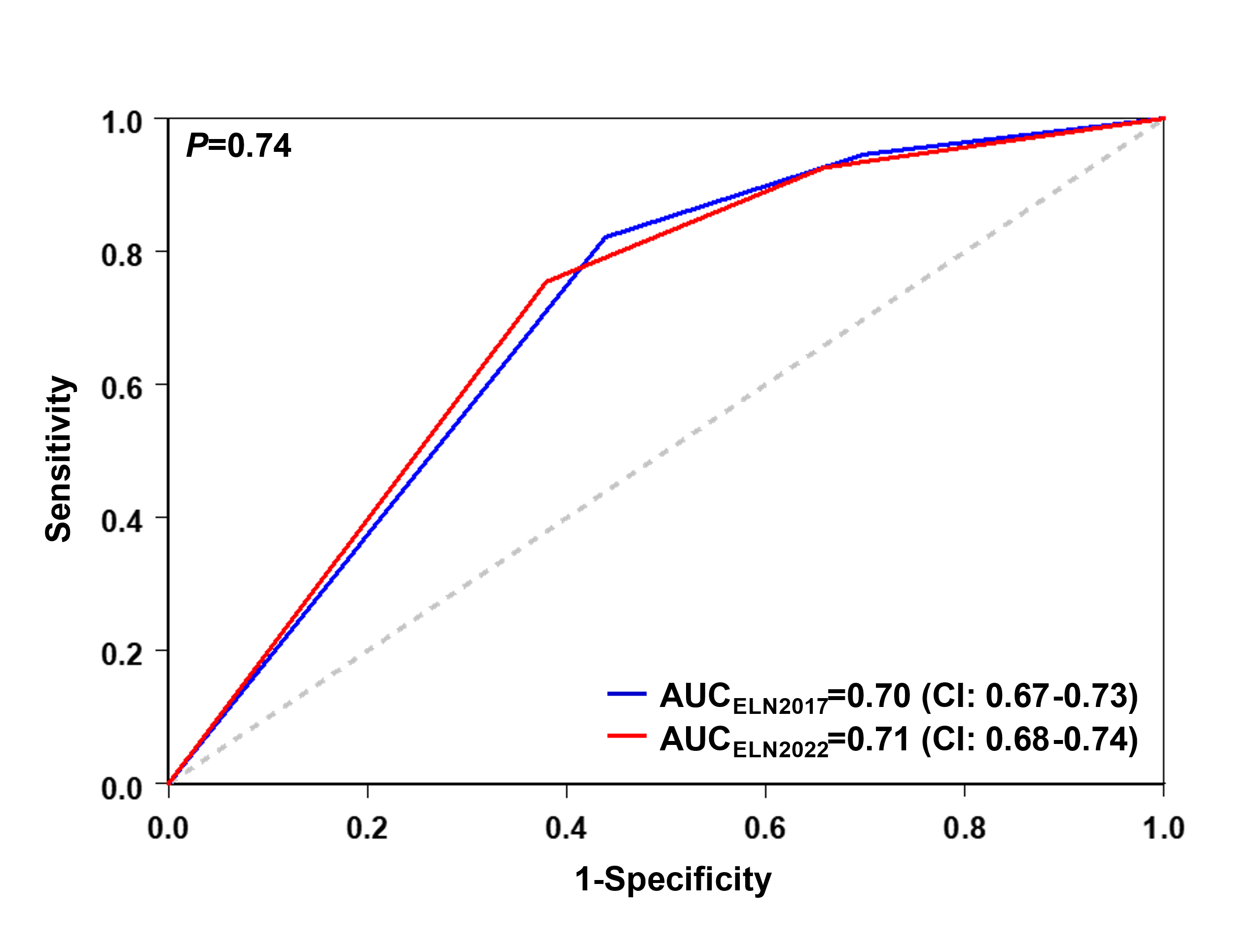

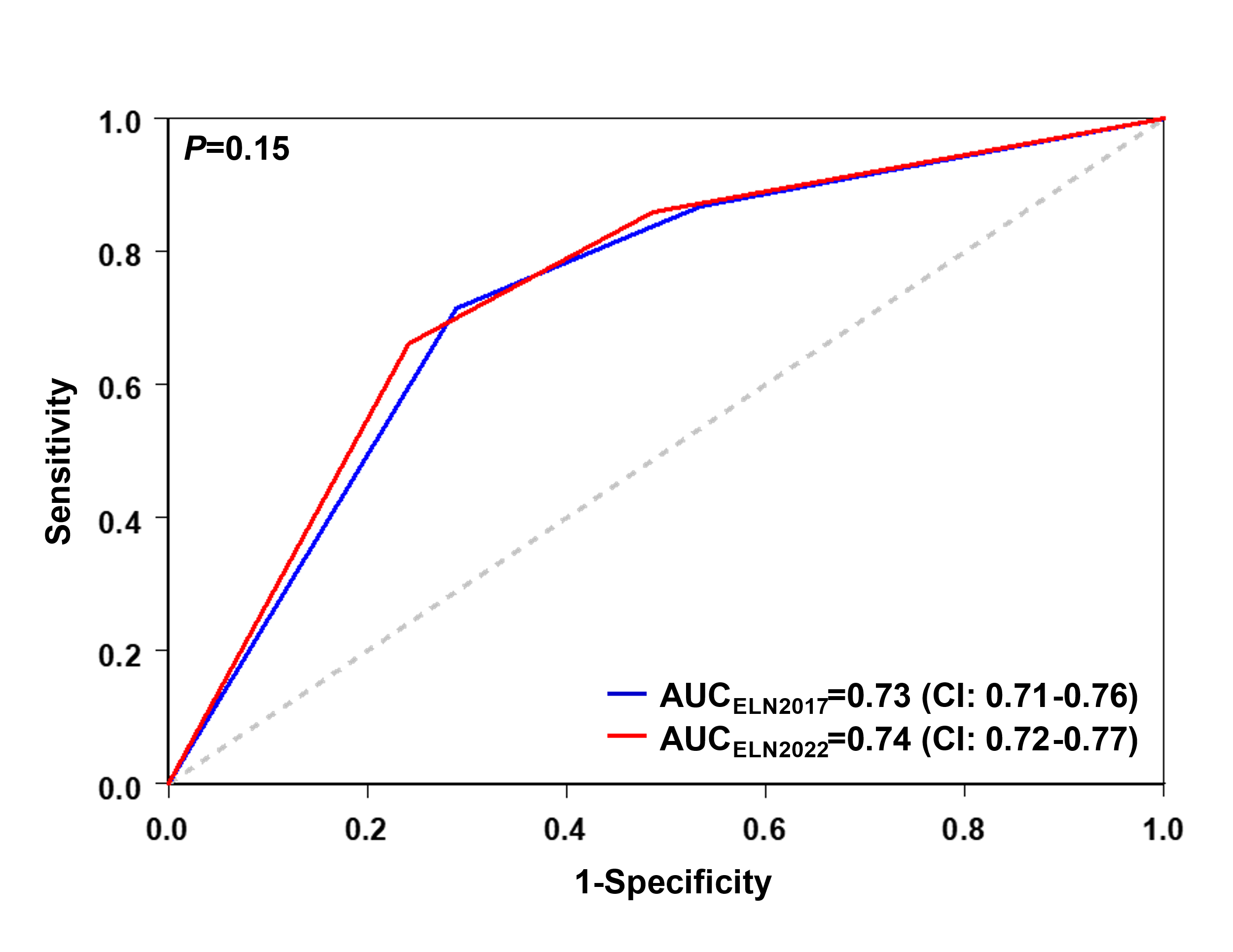


# Supplementary Figure 3. Outcome of younger (aged <60 years) Black and Hispanic patients with *de novo* acute myeloid leukemia categorized into the three genetic-risk groups according to the 2022 European LeukemiaNet recommendations. a Disease-free survival and b overall survival of younger Black patients. c Disease-free survival and d overall survival of younger Hispanic patients.

**a b**


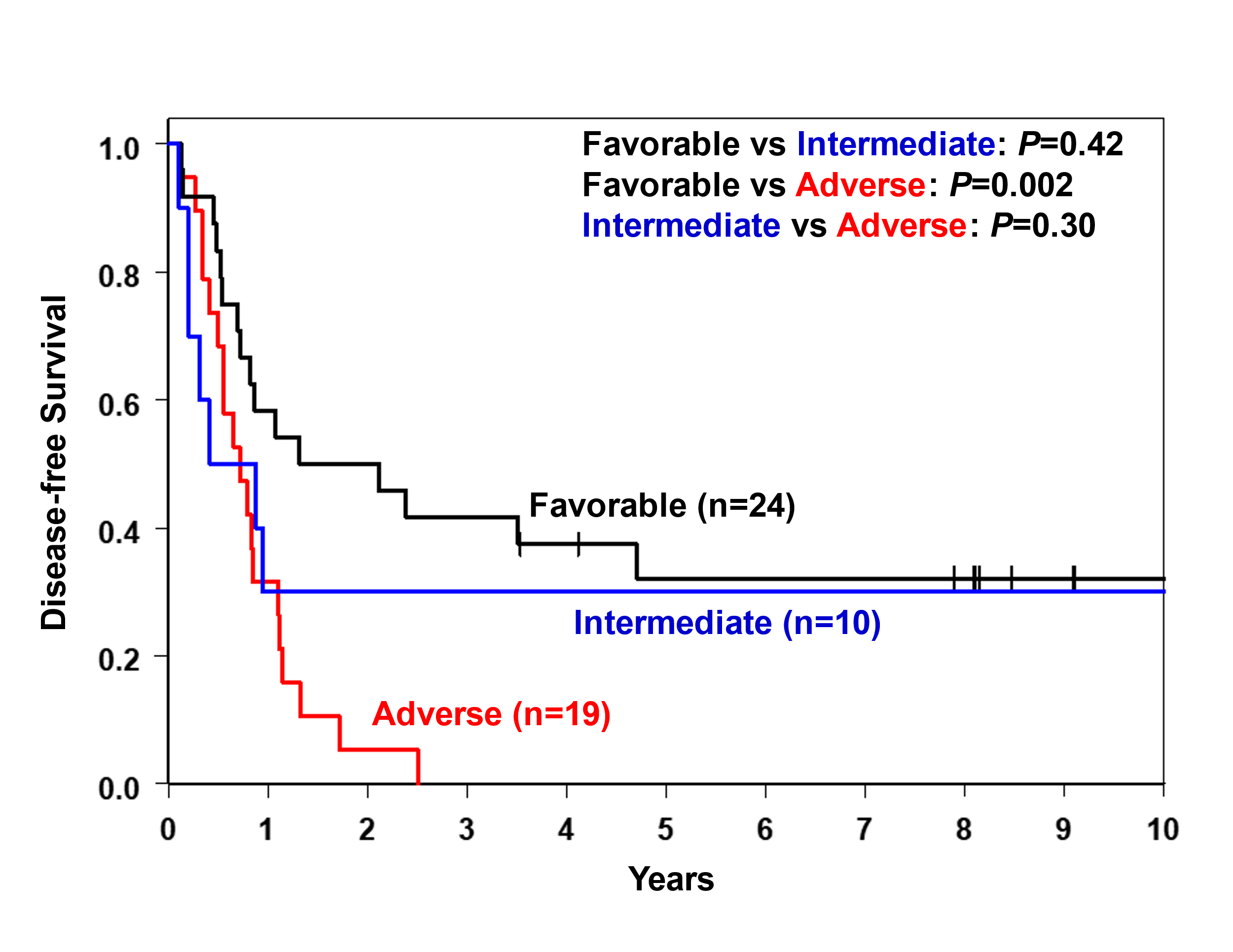

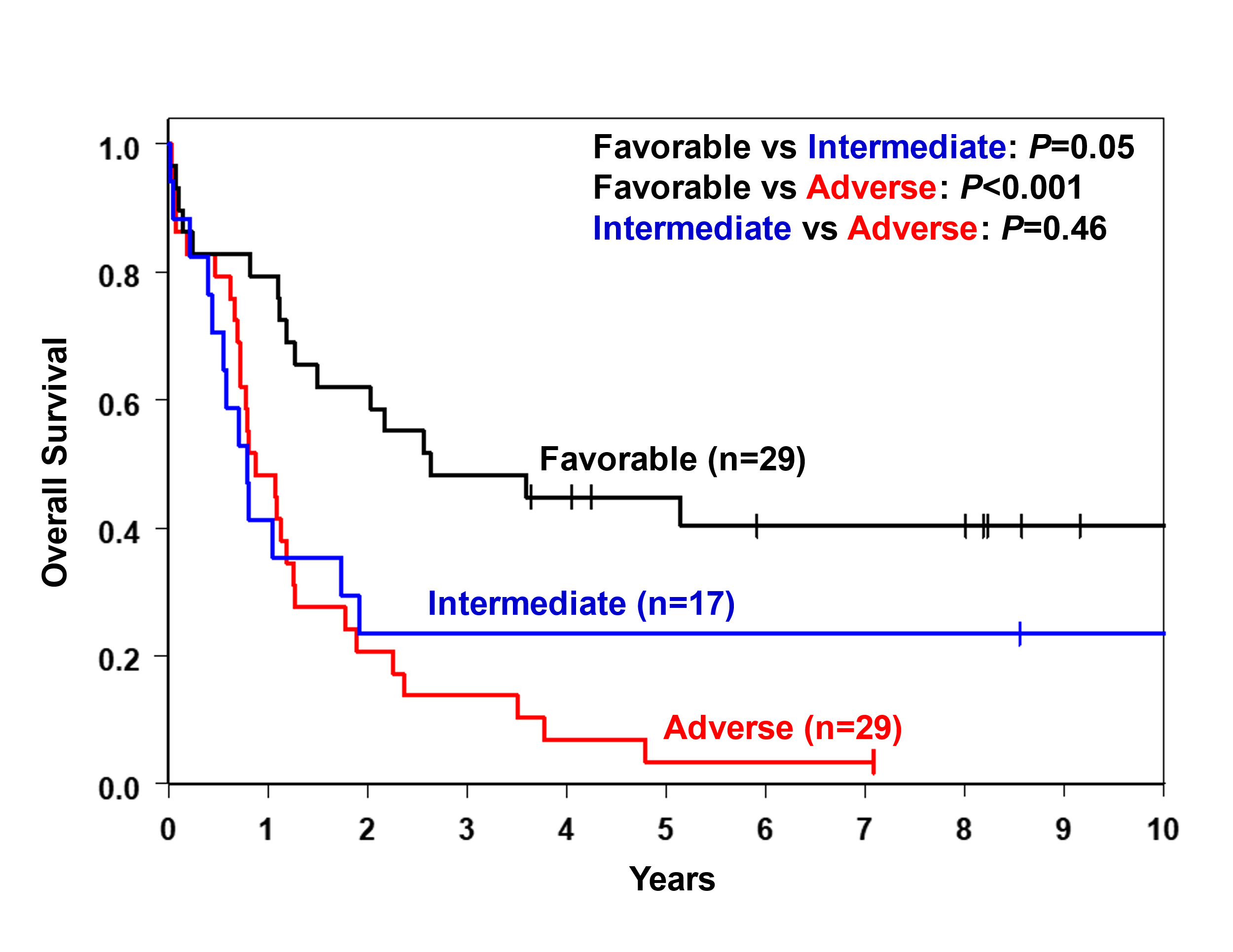


**c d**


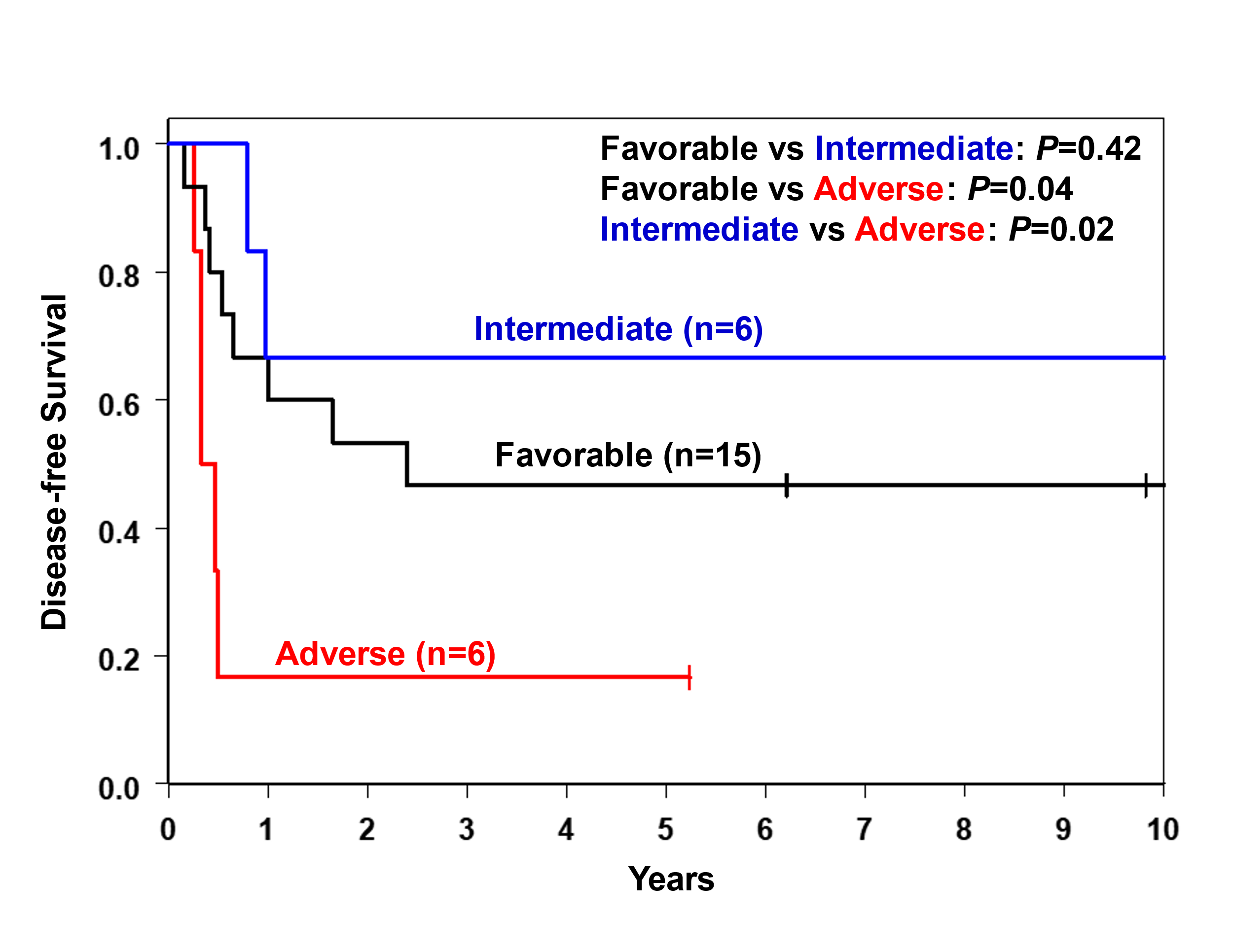

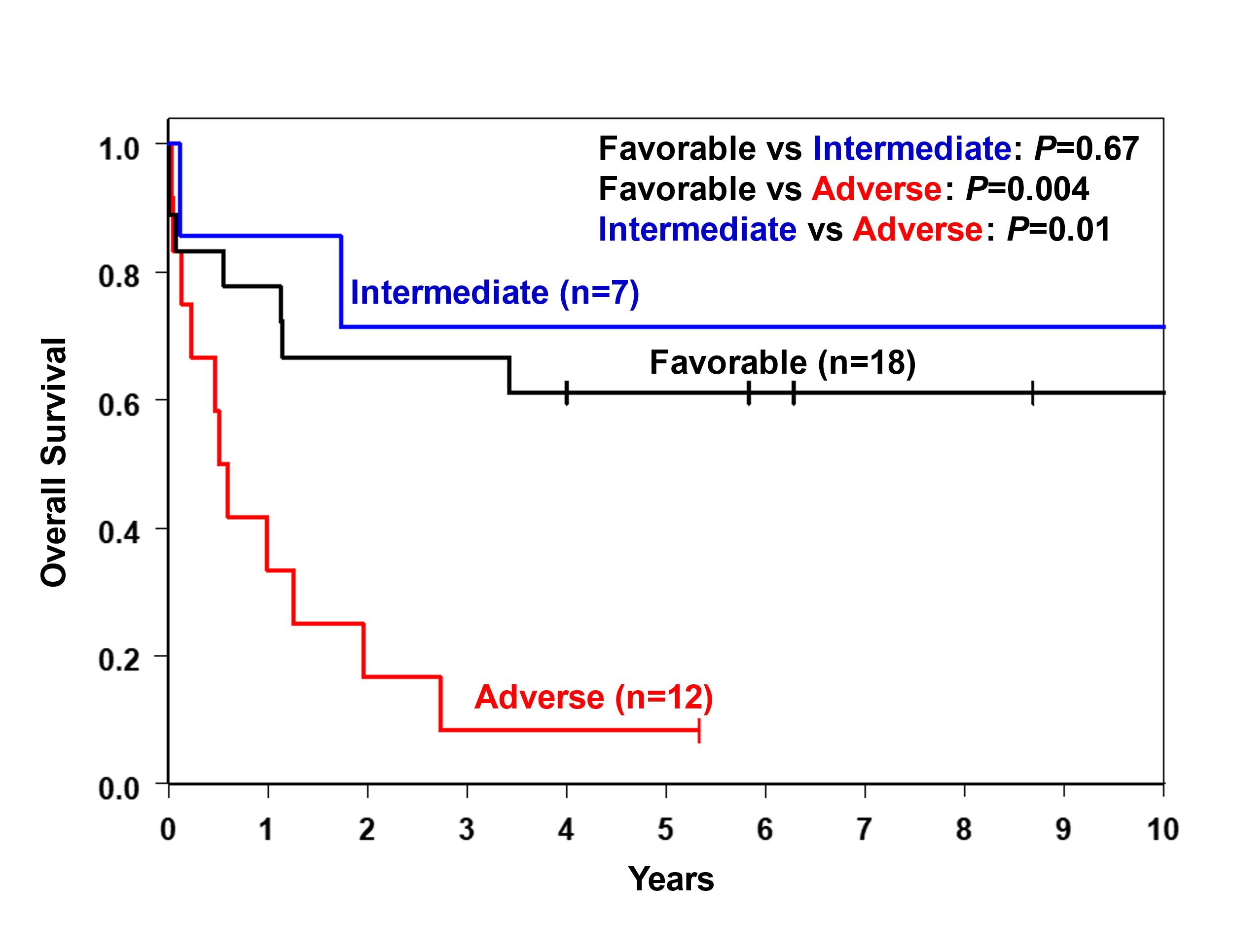


# Supplementary Figure 4. The receiver operating characteristic (ROC) curves illustrating the ability of 2022 European LeukemiaNet (ELN) genetic‑risk classification and 2017 ELN genetic‑risk classification to predict outcome in younger (aged <60 years) patients with AML. a Achievement of complete remission, b relapse rates, c 3‑year disease-free survival rates and d 3‑year overall survival rates.

**a b**

**
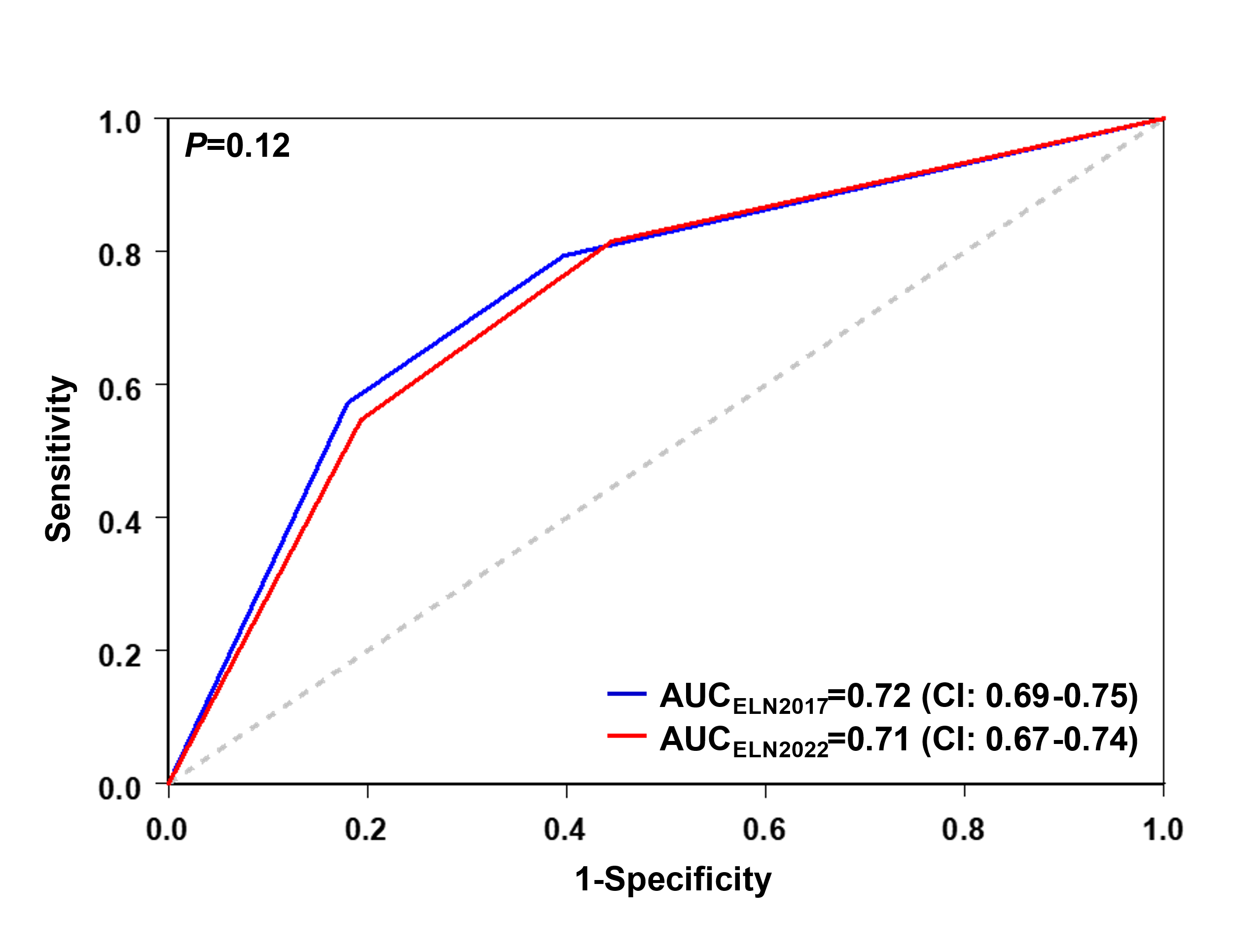

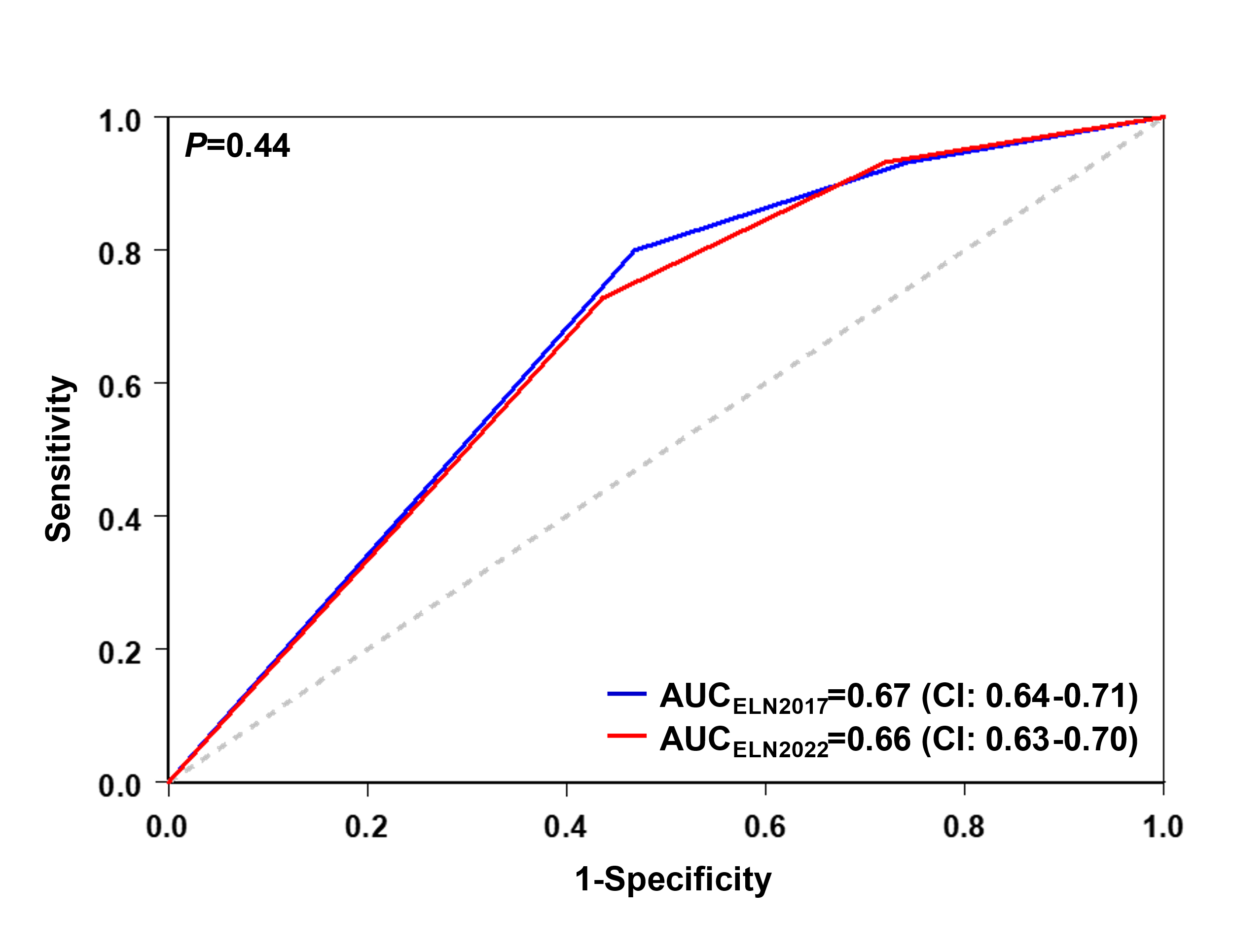
**

**c d**

**
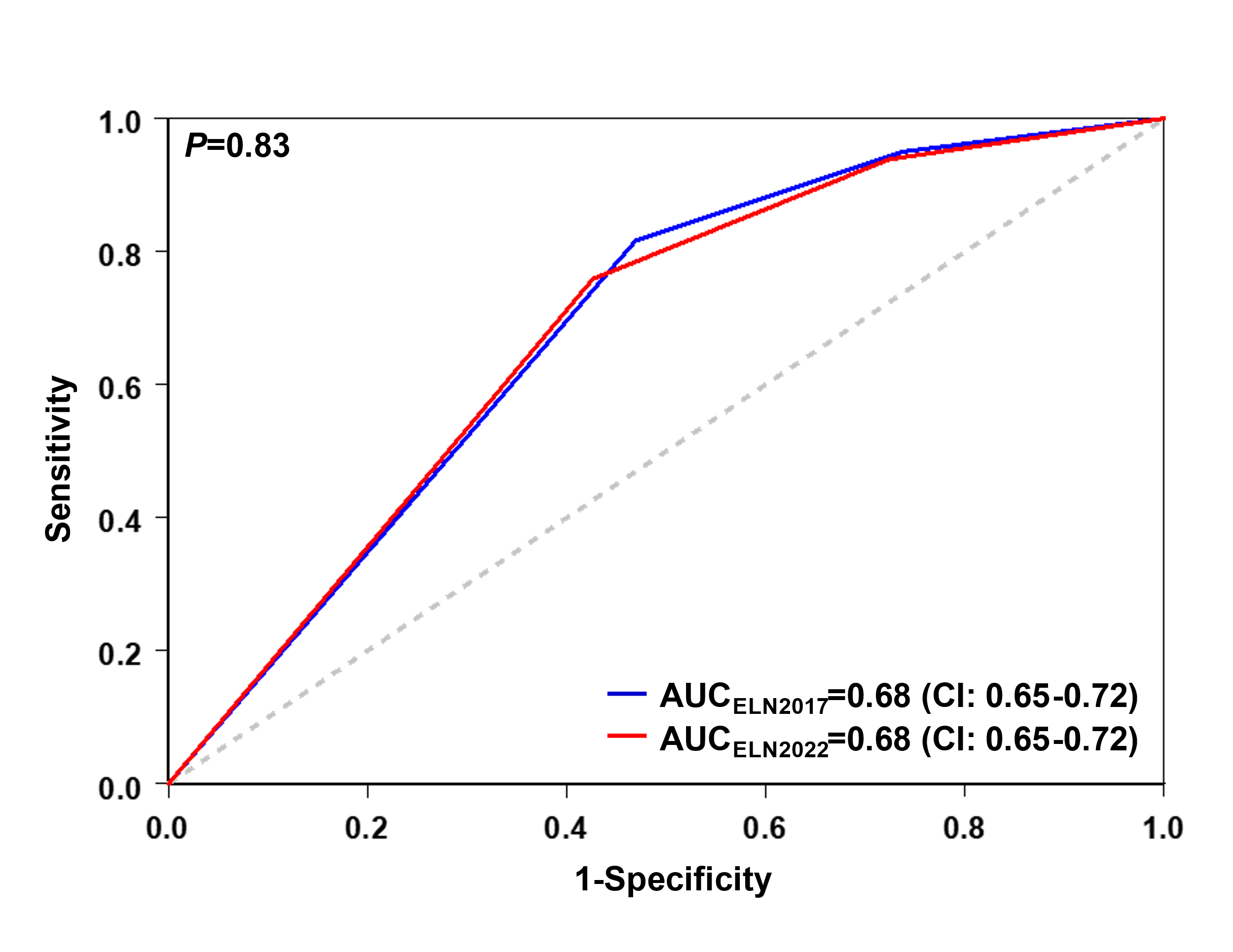
**
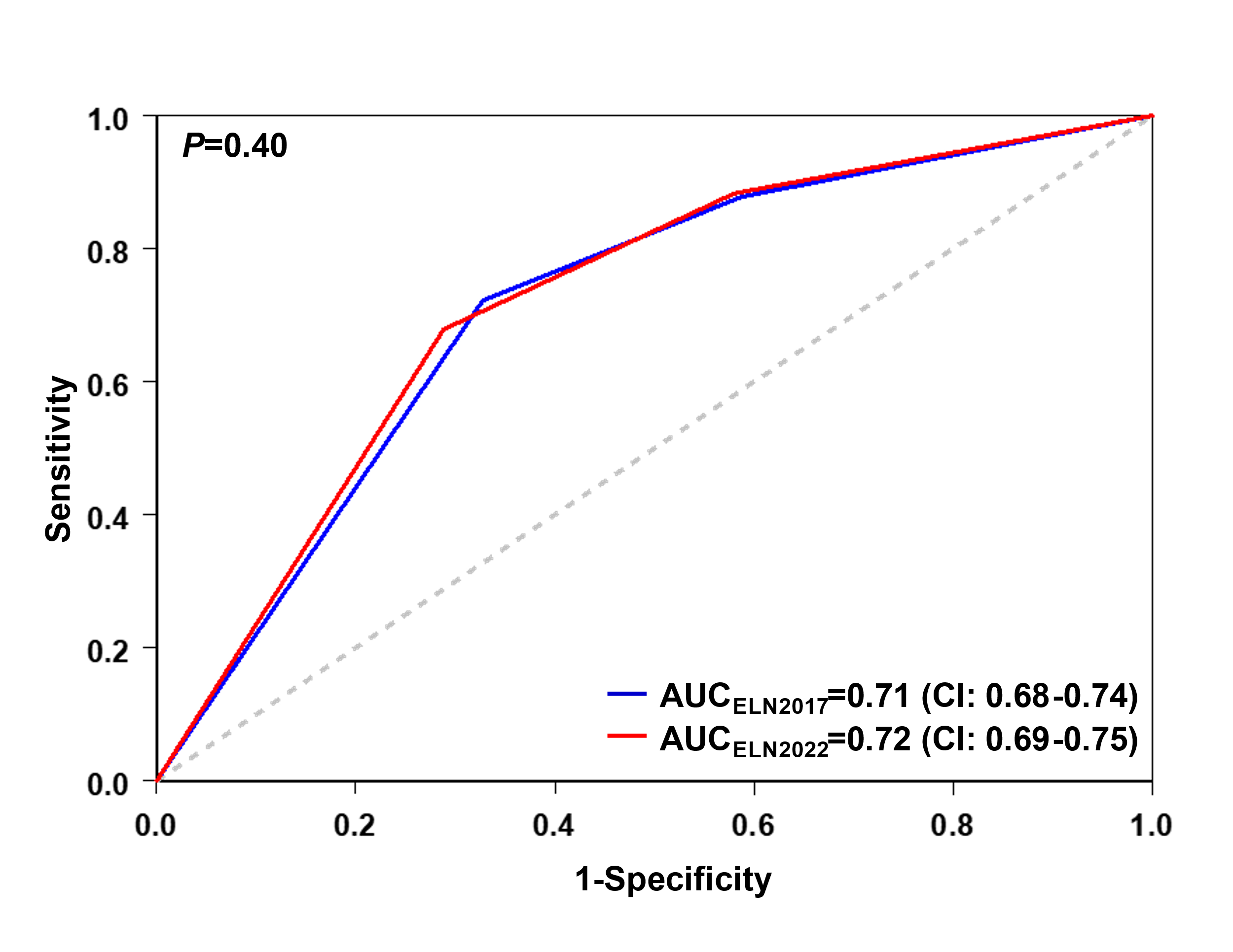


# Supplementary Figure 5. The receiver operating characteristic (ROC) curves illustrating the ability of 2022 European LeukemiaNet (ELN) genetic‑risk classification and 2017 ELN genetic‑risk classification to predict outcome in older (aged ≥60 years) patients with AML. a Achievement of complete remission, b relapse rates, c 3‑year disease-free survival rates and d 3‑year overall survival rates.

**a b**


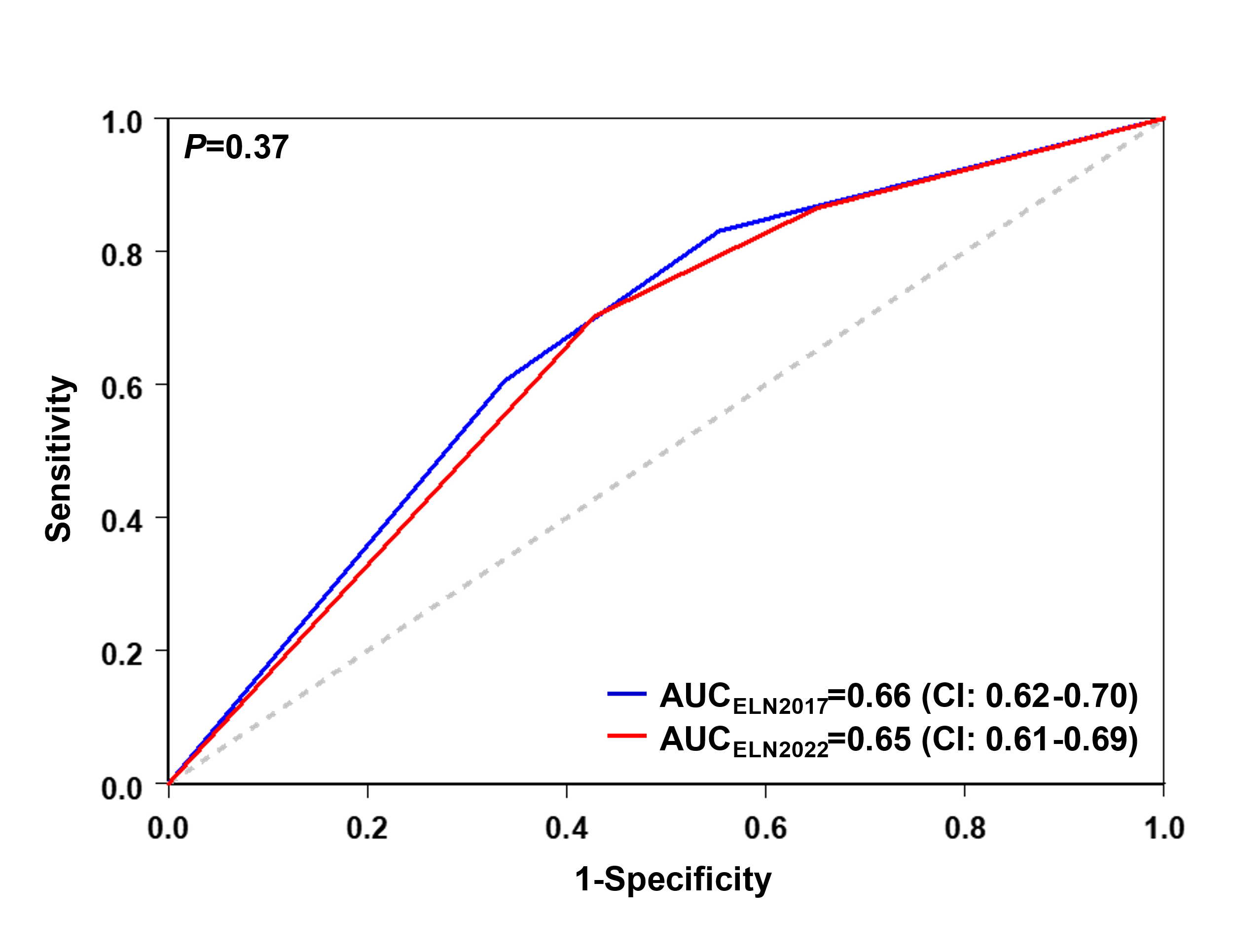
  **
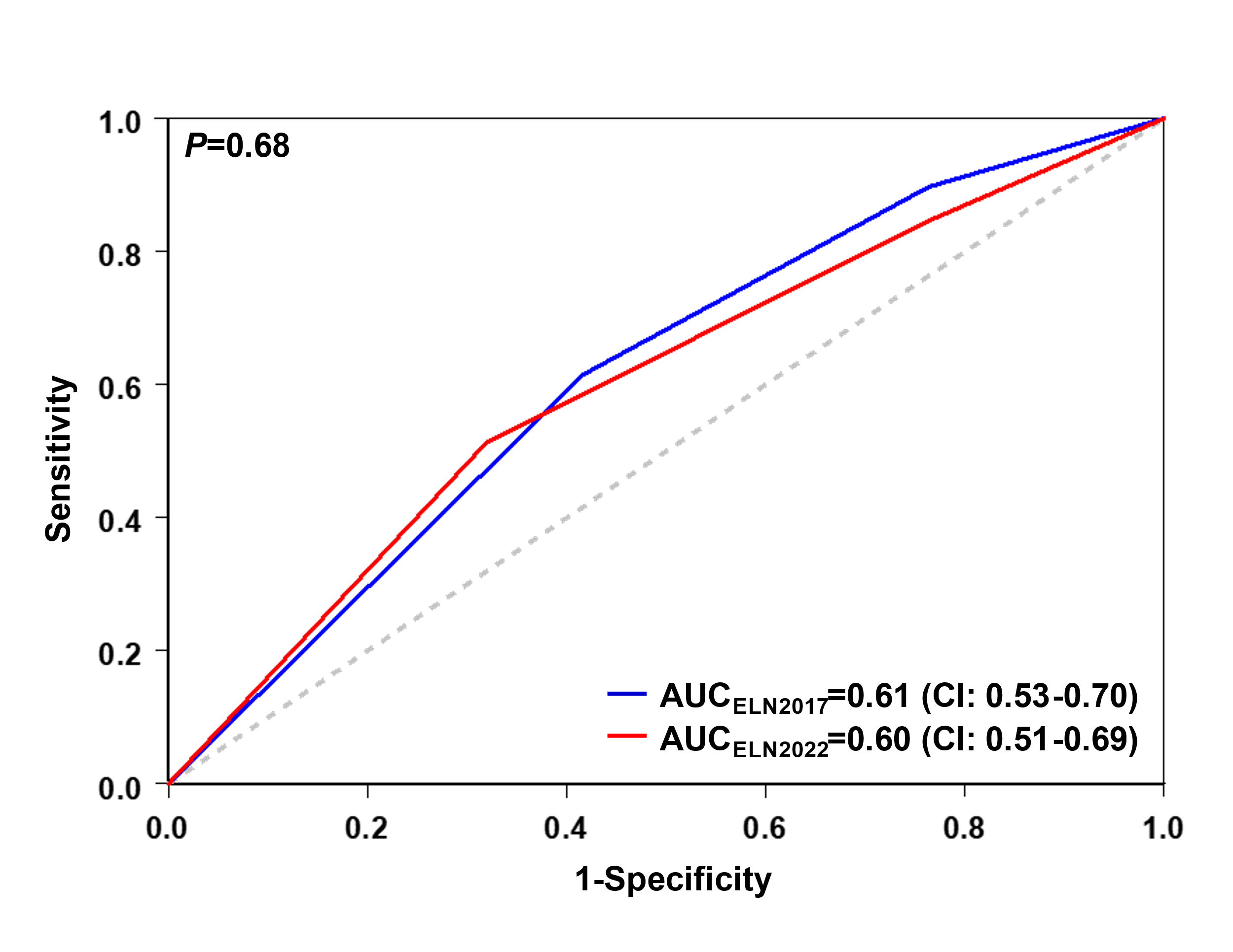
**

**c d**


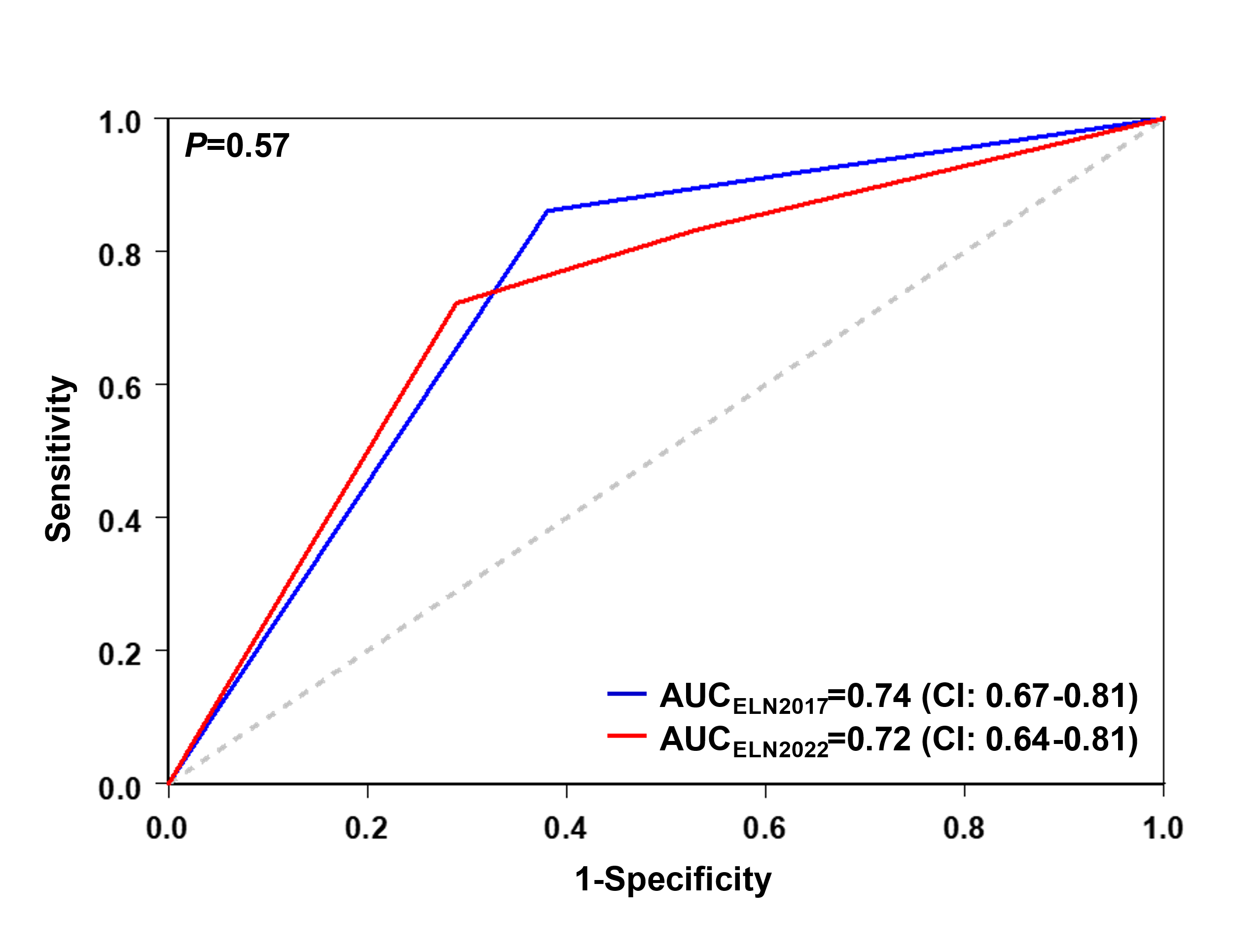
  **
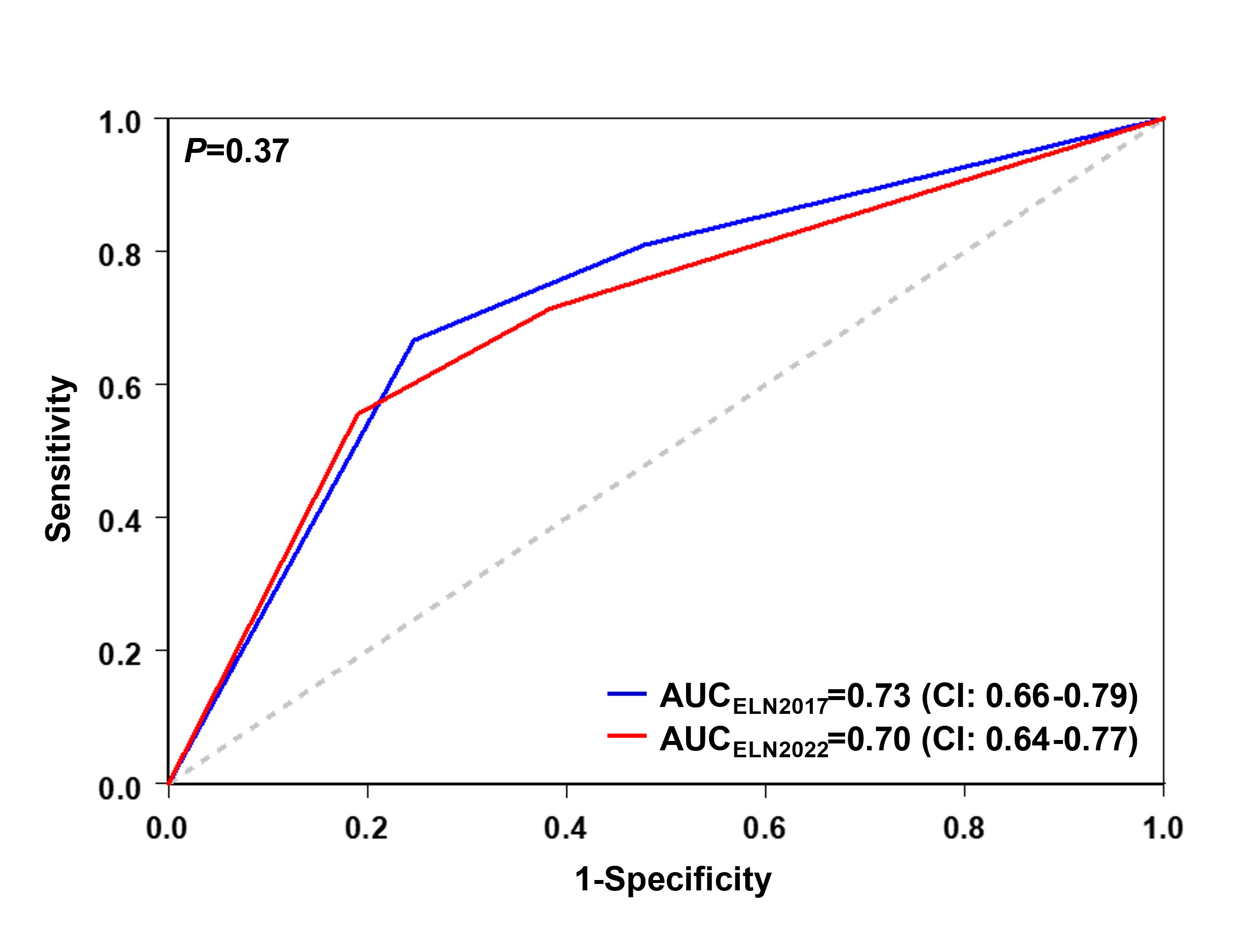
**

**Supplementary Figure 6. Outcomes of patients with *de novo* acute myeloid leukemia categorized according to the presence or absence of myelodysplasia-related mutations. a** Disease-free survival and **b** overall survival of patients with CBF-AML and myelodysplasia-related mutations and of patients with CBF-AML who do not carry myelodysplasia-related mutations. **c** Disease-free survival and **d** overall survival of patients with *CEBPA*^bZIP^ mutations and myelodysplasia-related mutations, and of patients *CEBPA*^bZIP^ mutations who do not have myelodysplasia-related mutations.

**a b**

**
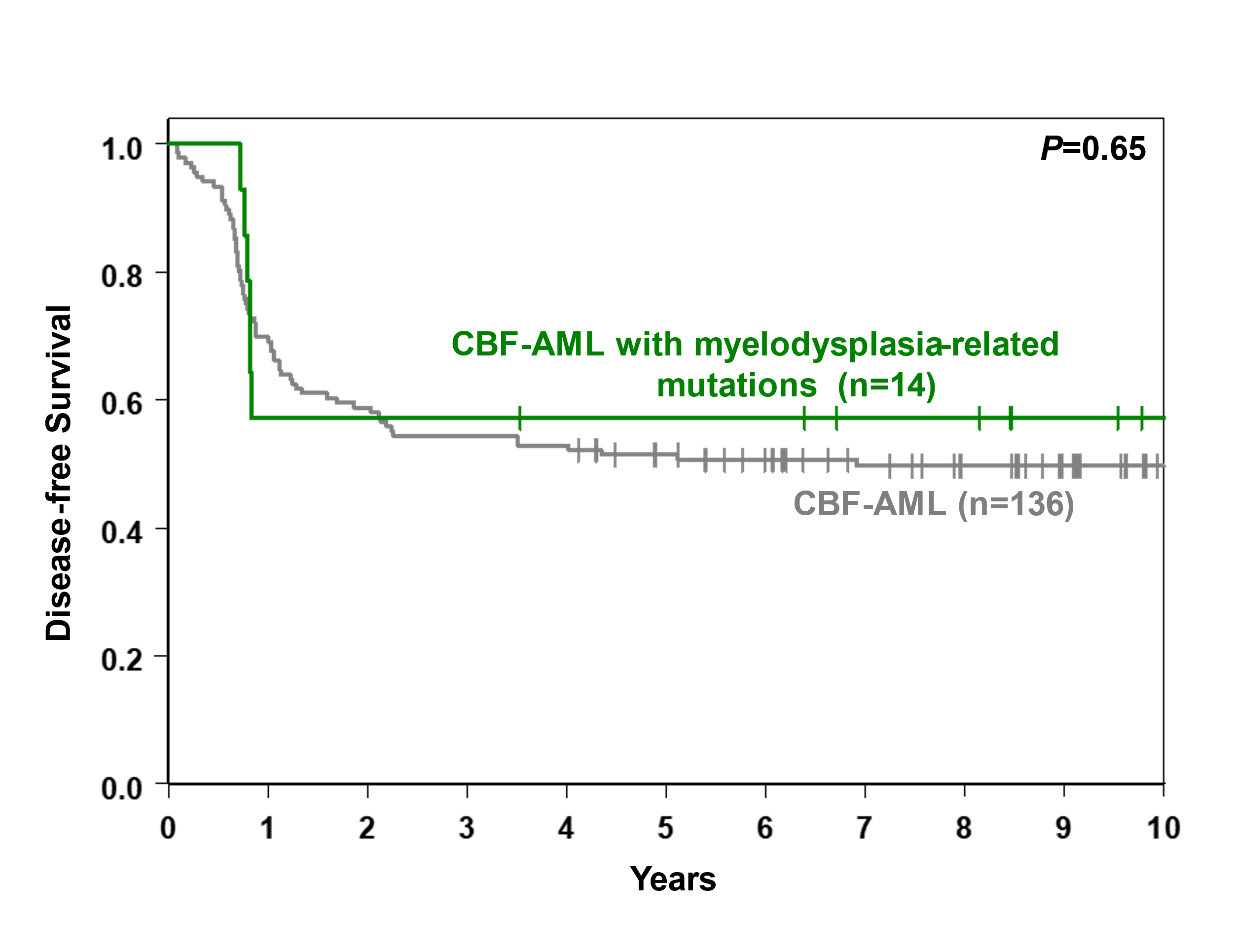

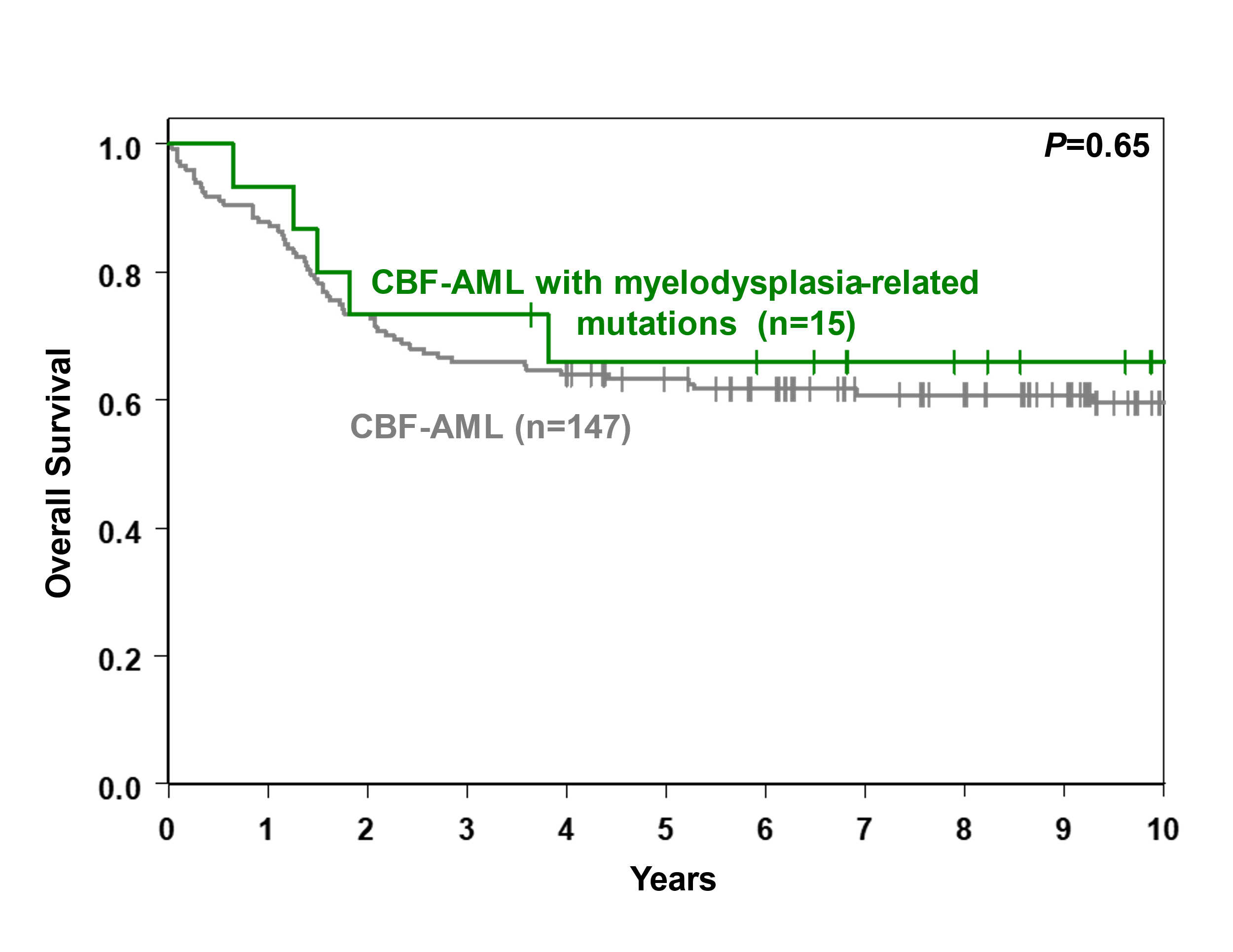
**

**c d**

**
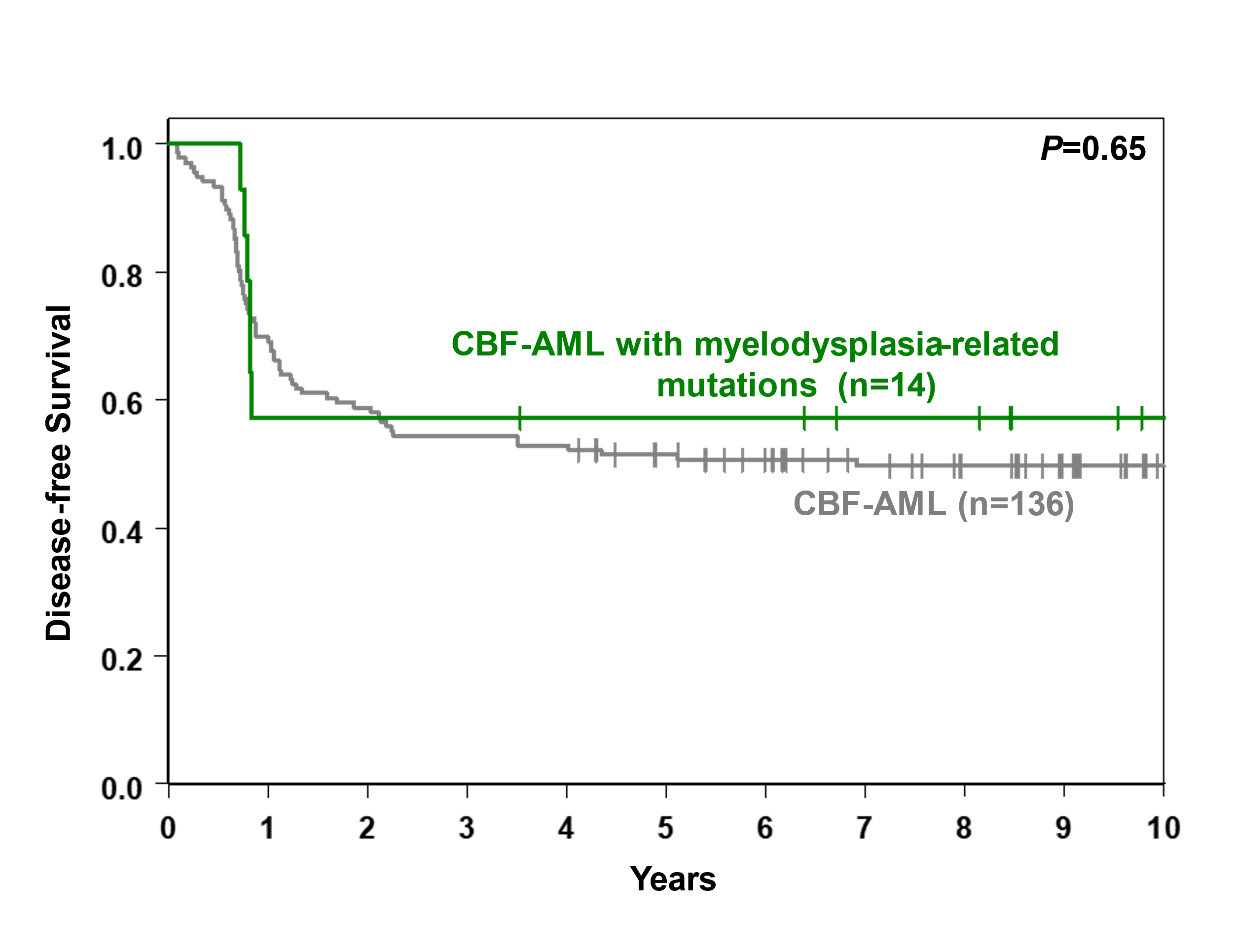

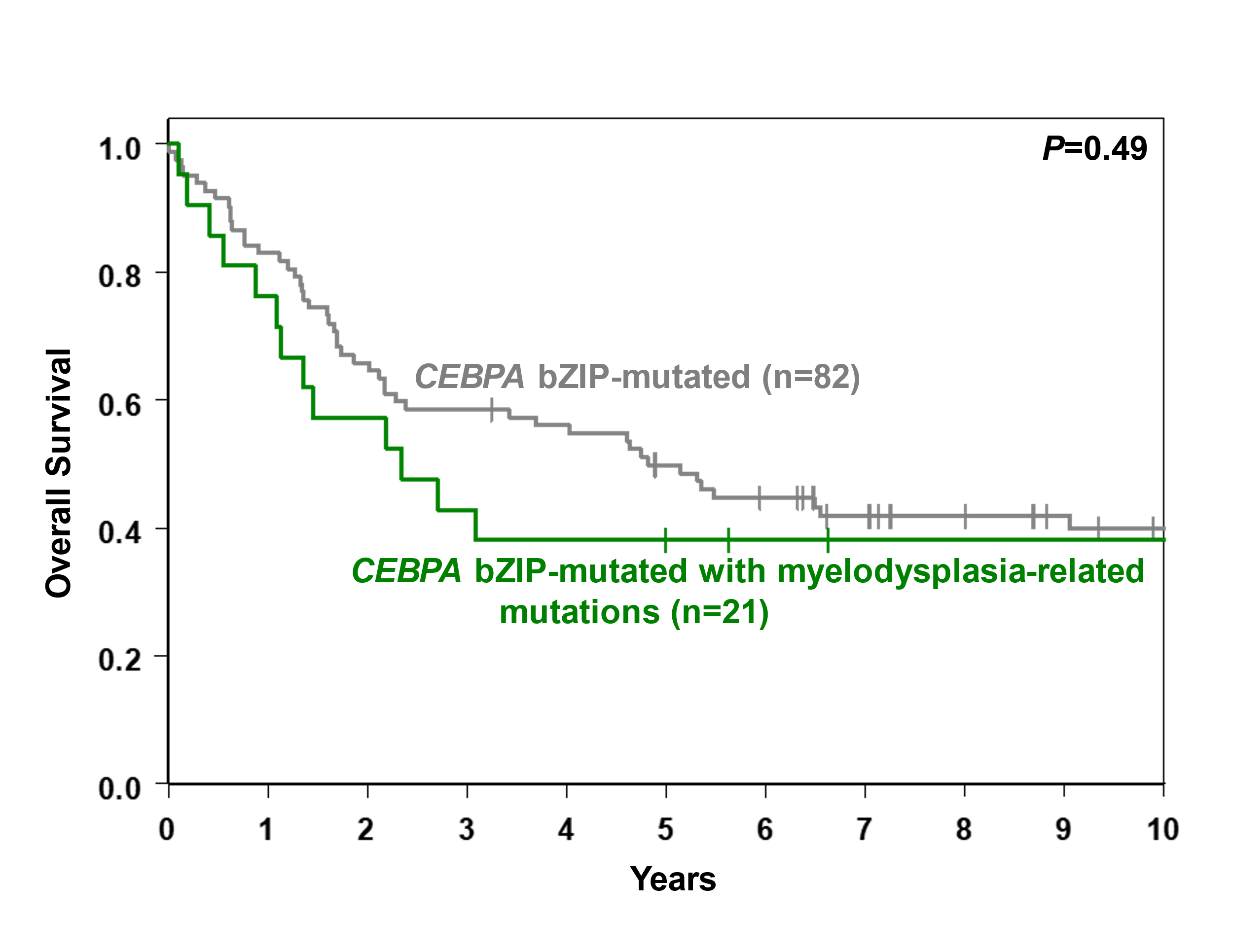
**

**Supplementary Figure 7. Outcomes of intermediate-risk patients with *de novo* acute myeloid leukemia harboring *FLT3*-ITD who were treated with midostaurin and those treated with chemotherapy only. a** Disease-free survival and **b** overall survival.

**a b**


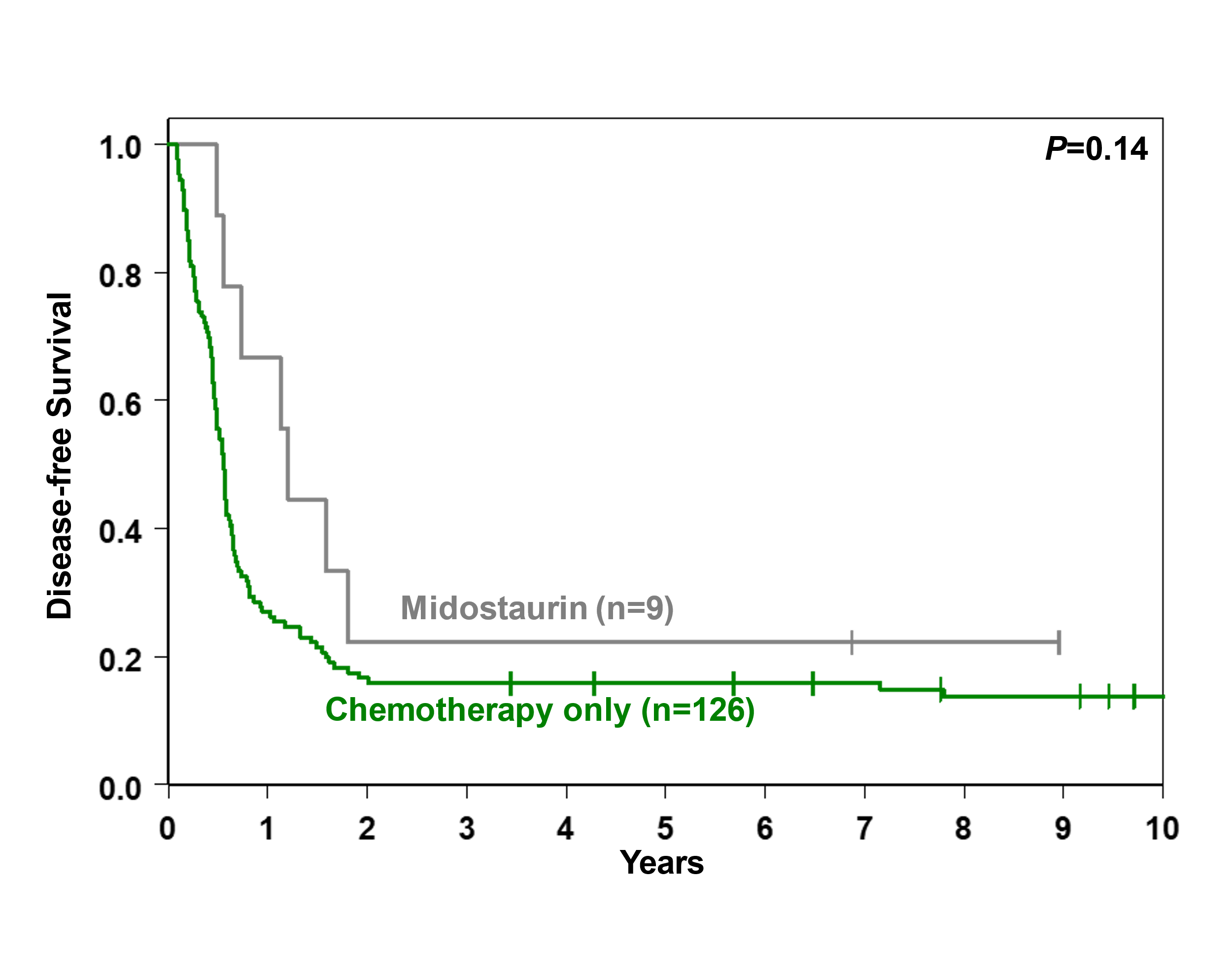

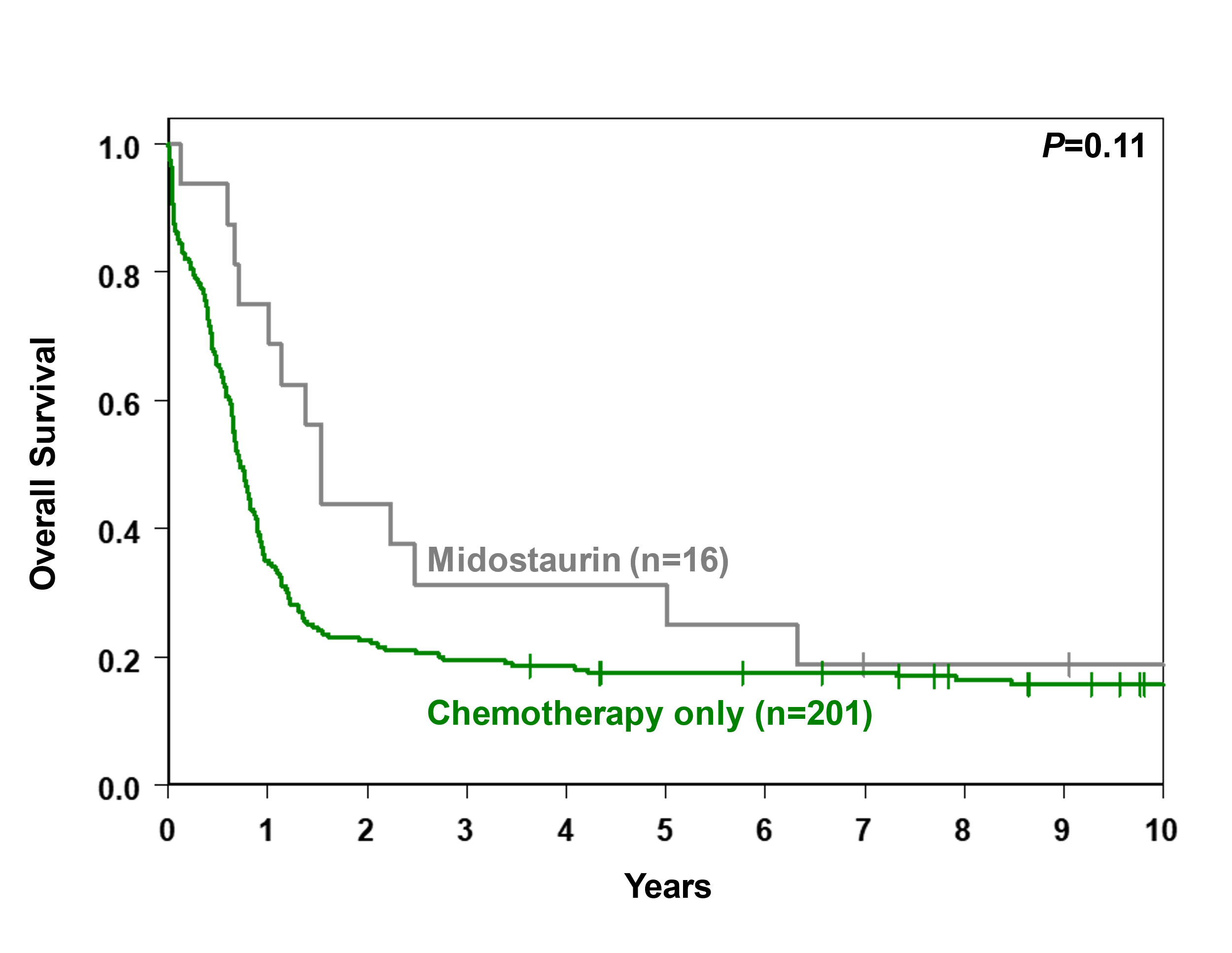


**Supplementary Figure 8. Kaplan-Meier curves illustrating outcome of patients with *de novo* acute myeloid leukemia and biallelic *CEBPA* mutations (both *CEBPA*^bZIP^ and non-bZIP *CEBPA* mutations) and of patients with in-frame *CEBPA*^bZIP^ mutations (both mono- and biallelic mutations).** **a** Disease-free and **b** overall survival. These patient groups cannot be formally compared because some patients are included in both groups. **c** Disease-free survival and **d** overall survival of patients with biallelic *CEBPA*^bZIP^ mutations, those with monoallelic *CEBPA*^bZIP^ mutations and patients with biallelic non-bZIP *CEBPA* mutations.

**a b**

**
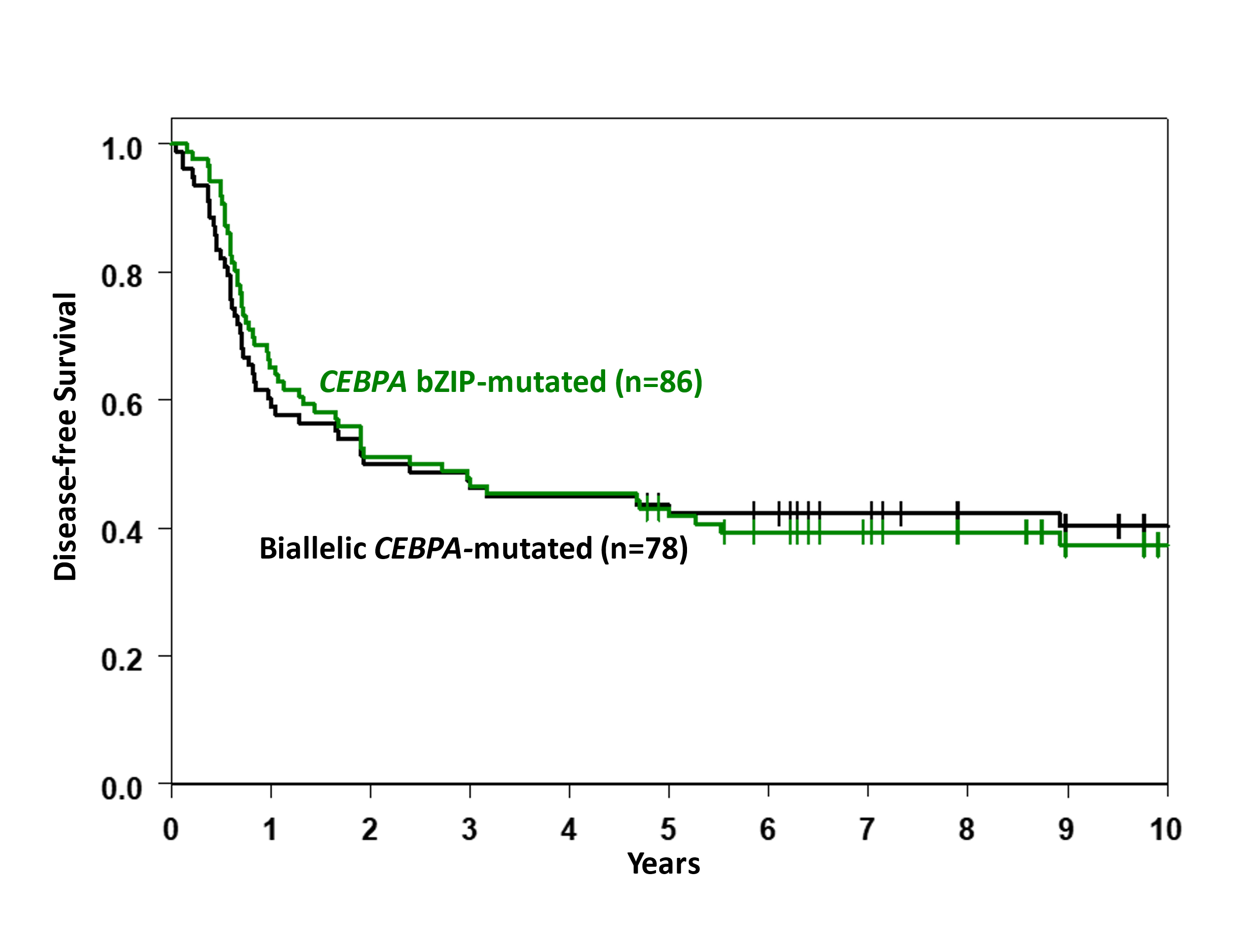

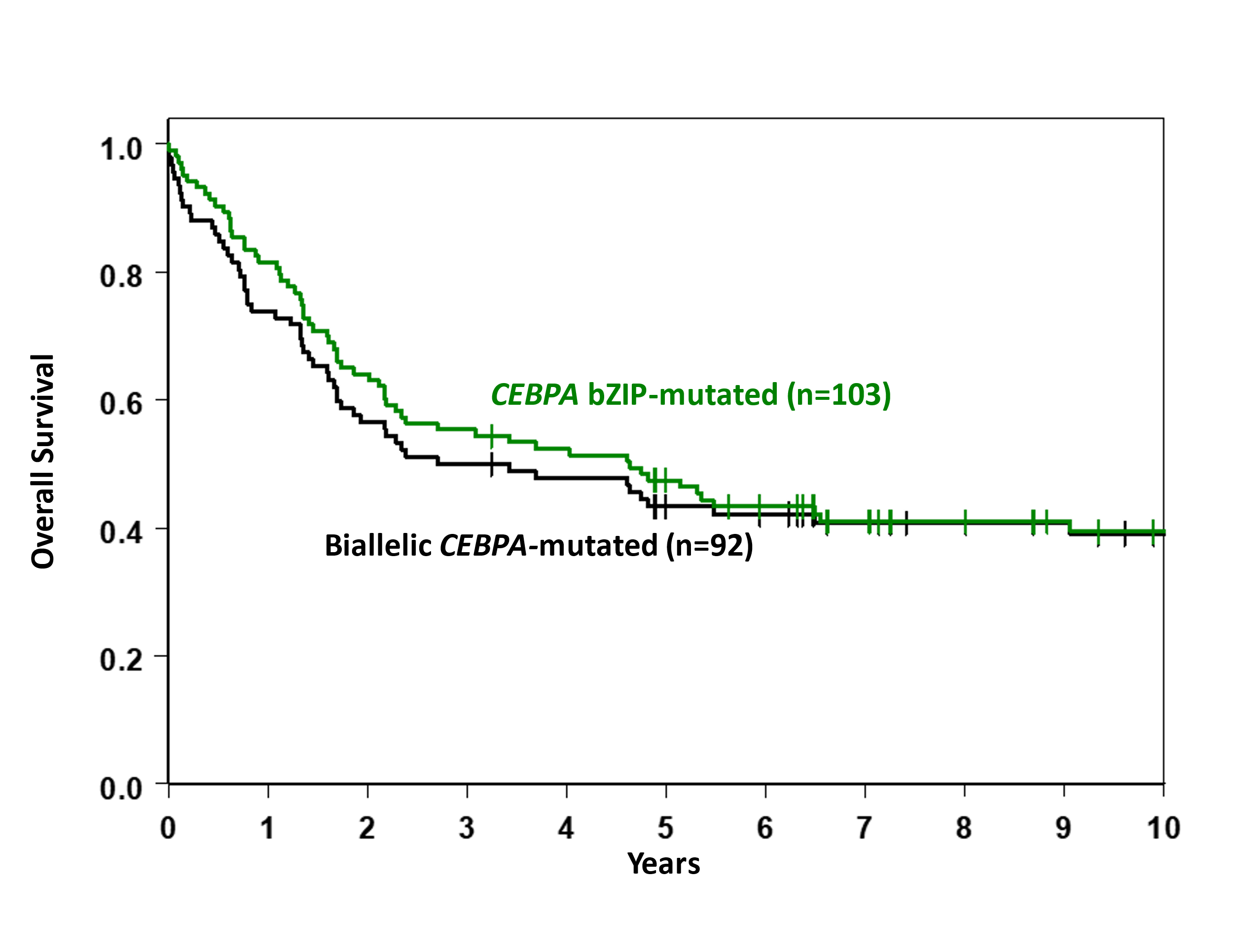
**

**c d**

**
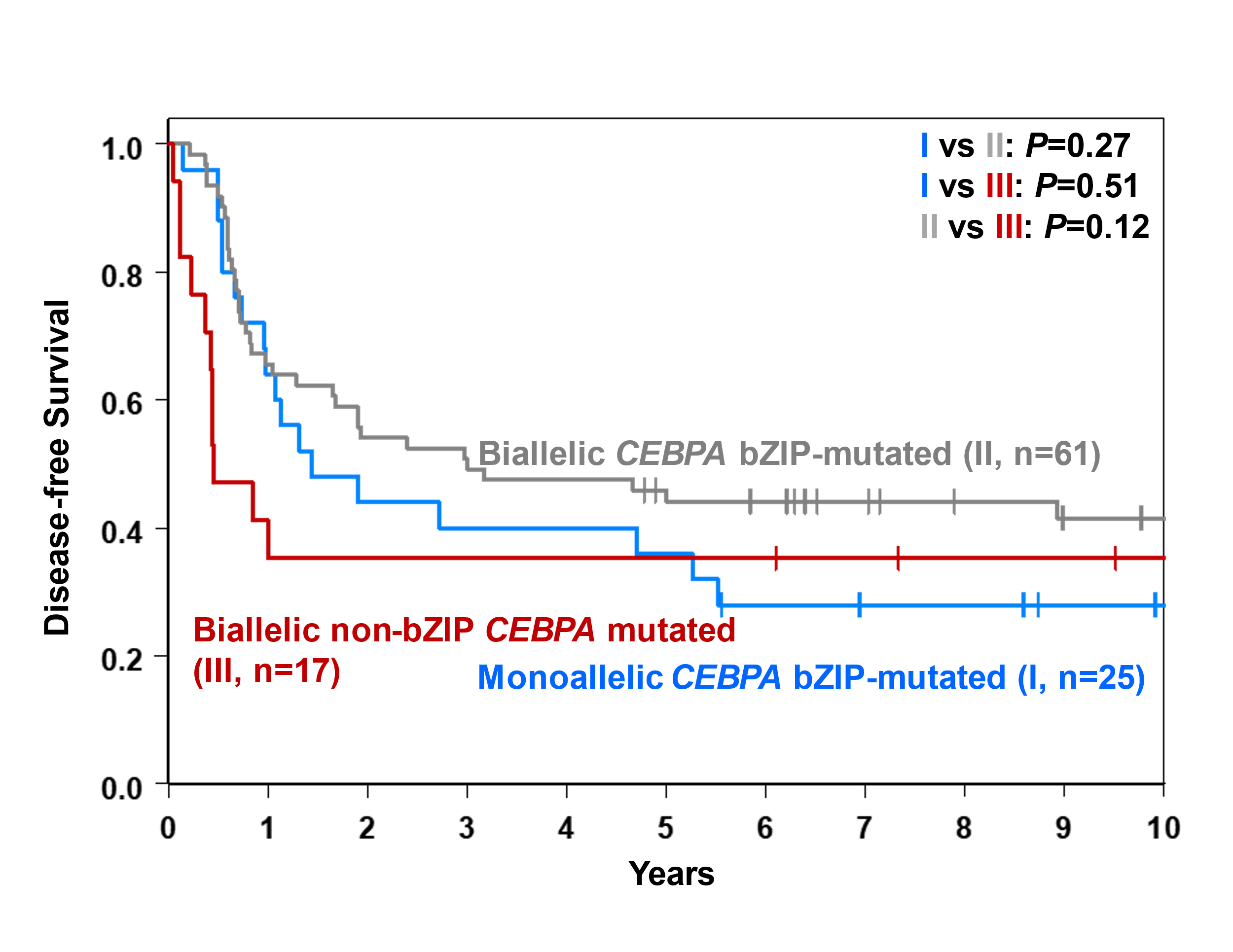

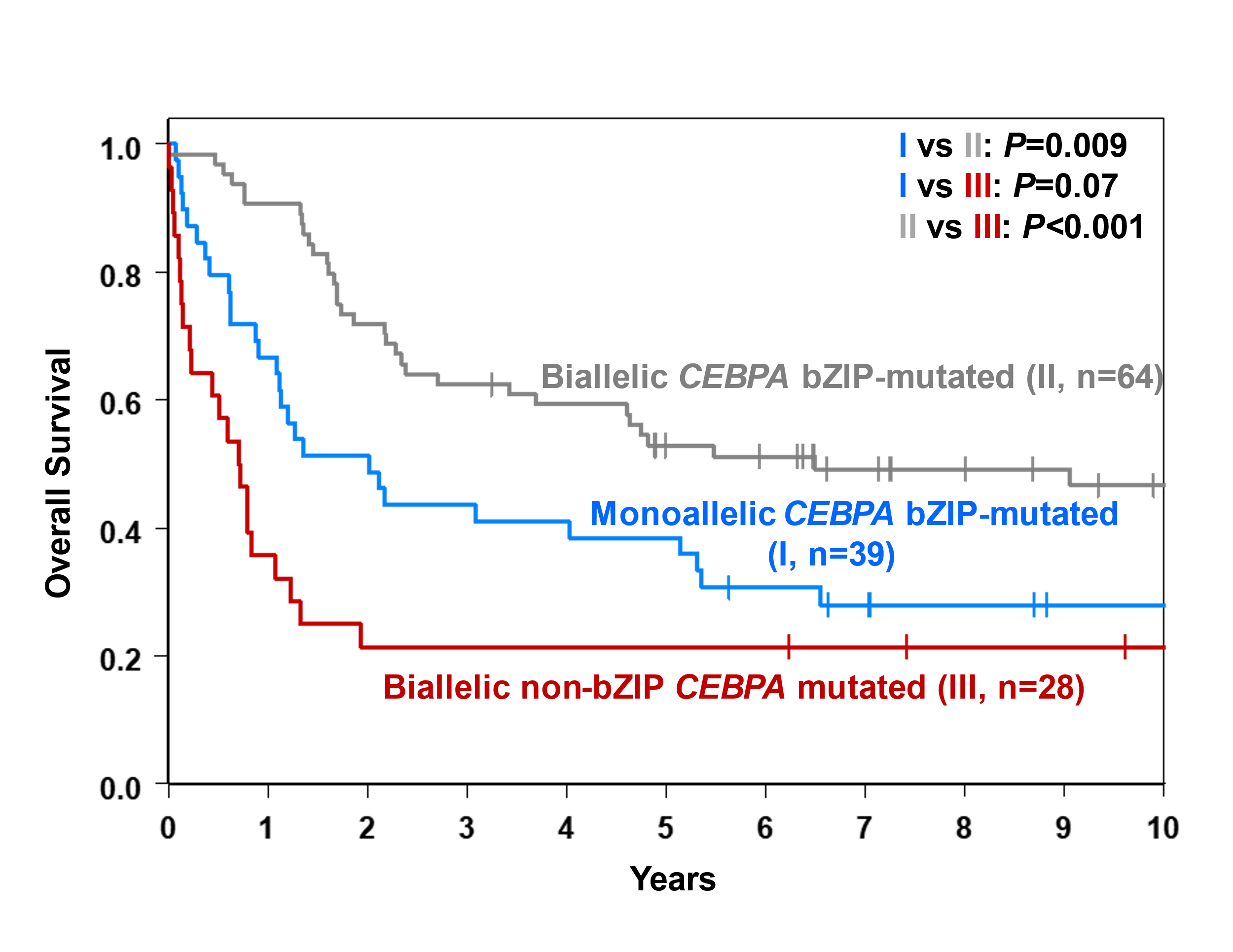
**
